# Narrative overview of animal and human brucellosis in Morocco: intensification of livestock production as a driver for emergence?

**DOI:** 10.1186/s40249-015-0086-5

**Published:** 2015-12-22

**Authors:** Marie J. Ducrotoy, Khaoula Ammary, Hicham Ait Lbacha, Zaid Zouagui, Virginie Mick, Laura Prevost, Ward Bryssinckx, Susan C. Welburn, Abdelali Benkirane

**Affiliations:** Division of Infection and Pathway Medicine, School of Biomedical Sciences, College of Medicine and Veterinary Medicine, The University of Edinburgh, Chancellor’s Building, 49 Little France Crescent, Edinburgh, EH16 4SB UK; Institut Agronomique et Veterinaire Hassan II, Rabat, Morocco; EU/OIE/FAO & National Reference Laboratory for Brucellosis, Animal Health Laboratory, Paris-Est, University/Anses, Maisons-Alfort, France; Avia-GIS, Risschotlei 33, BE-2980 Zoersel, Belgium

**Keywords:** Brucellosis, Morocco, Cattle, Small ruminant, Camel, Human, Control, Epidemiology, Surveillance, Emergence

## Abstract

Brucellosis is one of the most widespread zoonoses in the world caused by several species of the genus *Brucella*. The disease, eradicated in many developed countries, is a re-emerging neglected zoonosis endemic in several zones especially in the Mediterranean region, impacting on human health and livestock production. A One Health approach could address brucellosis control in Morocco but scarcity of reliable epidemiological data, as well as underreporting, hinders the implementation of sustainable control strategies. Surveillance and control policies implemented by the Moroccan government in domestic animals (cattle and small ruminants) in the last few decades are assessed for disease impact. This study considers the origins of animal brucellosis in Morocco and the potential for emergence of brucellosis during a shift from extensive to intensive livestock production.

**Electronic supplementary material:**

The online version of this article (doi:10.1186/s40249-015-0086-5) contains supplementary material, which is available to authorized users.

## Introduction

Brucellosis is one of the most widespread zoonoses caused by several species of the genus *Brucella* [1–3]. Presently, the genus includes 11 nominal species [4], among which *B. melitensis* and *B. abortus* are the most economically important and cause disease in cattle and small ruminants respectively. Brucellae show host preference but are not host specific, and spillover can occur when different host species are managed together or share grazing grounds and water sources*.* The disease, eradicated in many developed countries, is a re-emerging neglected zoonosis endemic in several zones, especially in the Mediterranean region [1, 5, 6], impacting on human health and livestock production [7, 8]. Across the African continent brucellosis is poorly documented [9–12] and under-reported in both human and animal populations [9].

Human brucellosis causes a flu-like illness with fever (which may be undulant), weakness, malaise, myalgia and weight loss. The disease is debilitating, often chronic and insidious and associated with serious complications (e.g., endocarditis, musculoskeletal lesions, spondylitis and neurobrucellosis) some of which are fatal if untreated. Clinical diagnosis is challenging and the disease is often misdiagnosed as malaria or other fevers [13]; for every case of brucellosis diagnosed, four are thought to go undetected [8]. Animals are the only significant source of human brucellosis; transmission occurs through direct contact with livestock or through the consumption of raw milk and dairy products. Brucellosis is an occupational hazard for veterinarians, abattoir workers and livestock keepers.

In livestock, brucellosis causes abortion, infertility in both male and female animals and reduced milk yields. Brucellae are excreted in vaginal secretions of infected females and are at their highest level immediately after abortion or birth; products of abortion and birthing materials are the main source of contagion, although vertical and sexual transmission and transmission through lactation also occurs. Extensive production systems exhibit low rates of disease transmission and lower disease burden, while intensification promotes transmission due to increased stocking densities, animal contacts and a higher birth index [1, 10, 12, 14, 15].

Control of brucellosis should be amenable to application of a ‘One Health’ approach [12, 16, 17] but under-reporting and a dearth of prevalence and incidence data impede implementation of appropriate control strategies. The cost effectiveness for brucellosis control has been demonstrated in a mass brucellosis vaccination programme in Mongolia [18]. In most developing nations, husbandry systems with poor veterinary inputs and the keeping of mixed species, close contact with humans, limited movement controls and lack of pasteurisation make brucellosis control difficult [19].

Morocco has an estimated population of 34 million people, mostly concentrated in the northwest [20] with 40 % involved in agriculture; 75 % of the rural poor derive their livelihoods from agriculture. Agriculture contributes 17 % of the GDP [21] and livestock accounts for 25–30 % of the agricultural GDP [22]. 18 % of farmers gain income solely from animal rearing, but livestock are kept by the majority as financial back-up to buffer against crop failure [23].

**Fig. 1:**
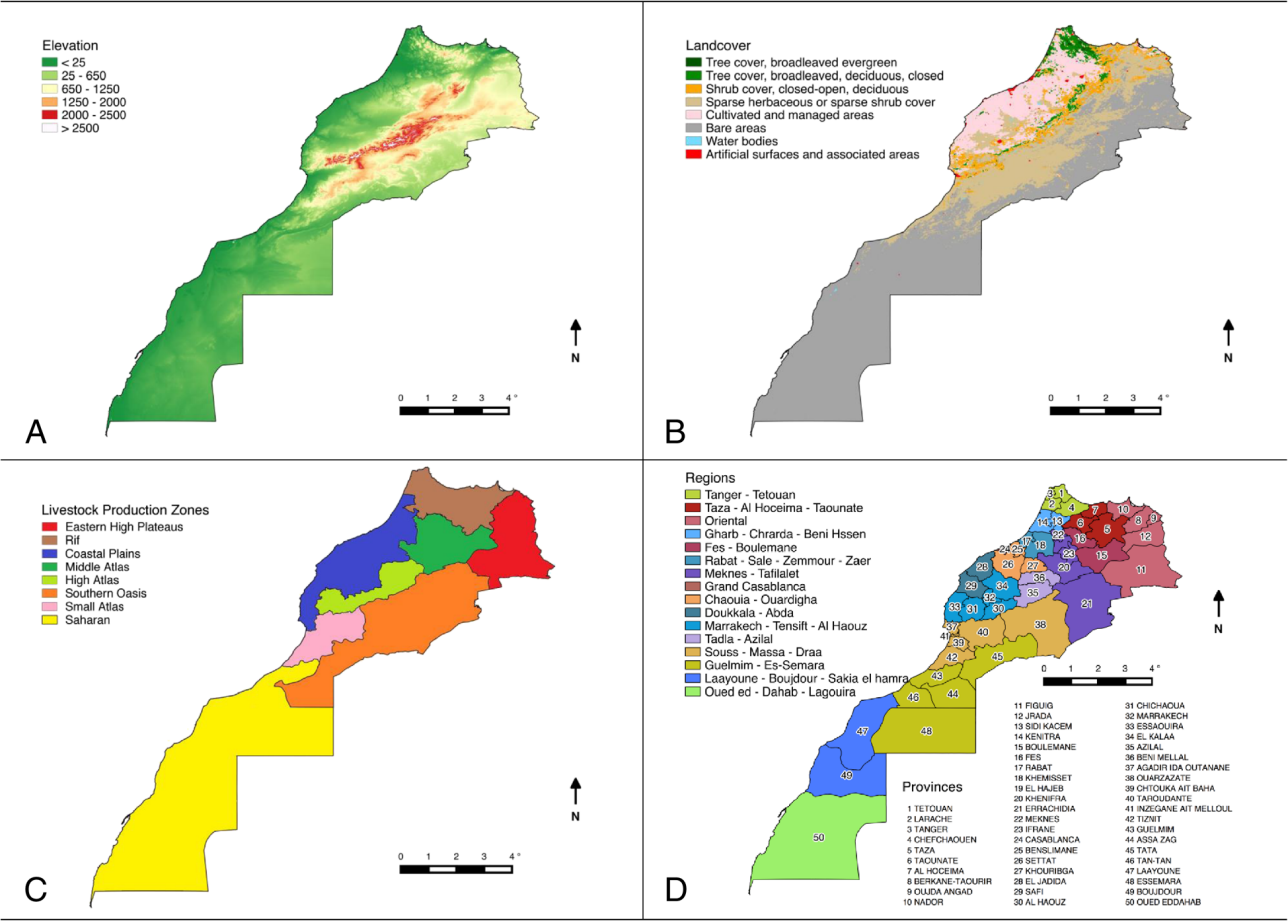
Terrain (**a**), landcover (**b**), agro-ecological/livestock production zones (**c**) and regions and provinces (**d**) of Morocco

Terrain, land cover, agro-ecological zones (Table 1), regions and provinces of Morocco are displayed in Fig. 1. Intensive agriculture is found mostly in irrigated areas along the Atlantic coast. Vast areas of steppe east of the middle Atlas and on the high eastern plateau are used as rangeland for extensive livestock production. Government estimates for 2014 put cattle, sheep, goat and camel populations at 3.23 million, 19.23 million, 6.15 million and 178,825 respectively [24] in line with the 3.17 million, 19.96 million, 6.24 million respectively reported by the FAO [25] (although the estimate for camels is substantially lower at 57,000).

**Table 1:** Main agro-ecological zones in Morocco

Zone	Dominant agriculture & production system
Eastern high plateau	Sheep and goat nomadic system shifting to more settled. Barley. Dairy cattle in irrigated areas.
Middle Atlas	Integrated crop and livestock (sheep, goats and some cattle) subsistence system. Summer transhumance of sheep flocks.
Rif, high Atlas, small Atlas, southern Oasis	Settled, diversified (crop livestock combinations), relatively intensive and usually irrigated. Forage production and conservation.
Coastal plains	Large-scale cereal cultivation associated with increasingly intensive sheep and cattle (dairy and beef) production. Irrigated perimeter and rainfed agriculture. Mix of subsistence and large farms.
Saharan	Cropping limited to irrigated areas. Dominance of extensive livestock production (goats, sheep and camels).

This review examines the evolution of the epidemiological situation of brucellosis in domestic ruminants and humans in Morocco and describes surveillance, control and livestock policies implemented by the Moroccan government. The origin and emergence of animal and human brucellosis in Morocco are discussed.

## Cattle brucellosis

### Cattle production systems and policies

**Fig. 2:**
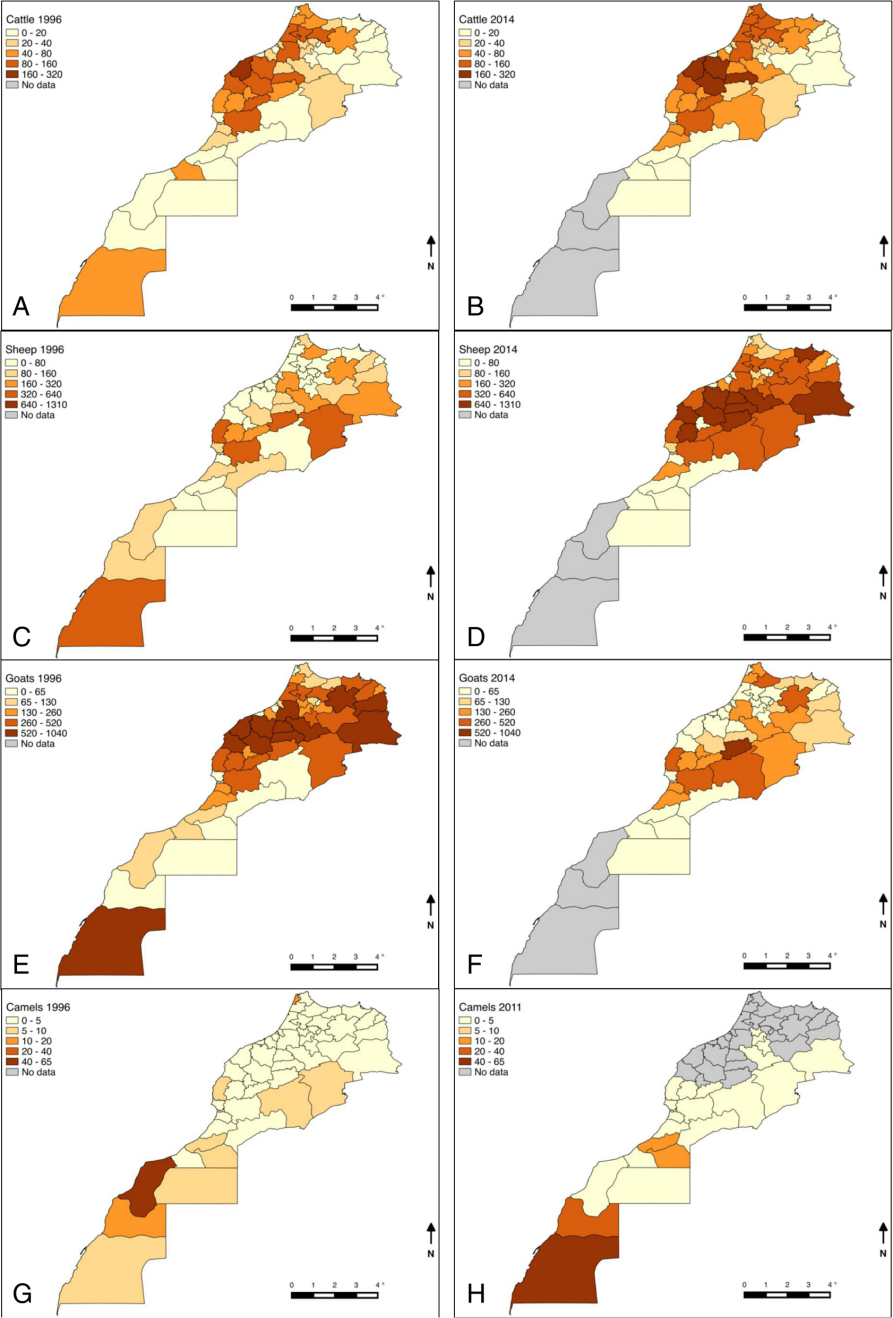
Distribution of cattle in 1996 (**a**) and 2014 (**b**); sheep in 1996 (**c**) and 2014 (**d**); goats in 1996 (**e**) and 2014 (**f**); and camels in 1996 (**g**) and 2011 (**h**)

Cattle are mostly distributed in the coastal plains (Fig. 2a, b) in three main systems of production: dairying (intensive), mixed (semi-intensive) and beef (extensive). Currently, 85 and 60 % of cattle in the intensive dairying and overall cattle sector respectively are imported breeds [26]. The dominance of imported cattle (compared with the dominance of local breeds prior to the 1960s) relates to government schemes to improve productivity by importation of European breeds.

Cattle production occurs mainly on mixed smallholder farms. Only 5 % of farms are specialised dairy farms [27]. 80 % of farmers who own less than 5 ha of land each keep more than 60 % of the cattle and 50 % of the sheep on only 25 % of the cultivable land [22]. Statistics from 1997 show the dominance of smallholder systems with 85, 14 and 1 % of all livestock keepers owning less than two, between three and six, and more the 11 cattle respectively [28].

The government implemented a Dairy Plan or ‘Plan Laitier’ in 1975 to establish intensive dairy production and meet increasing demands for milk for urban dwellers [27], targeting coastal, irrigated and peri-urban zones [29]. This increased fresh milk production from 580 million in 1975 to 2.5 billion litres per year by 2012 [26].

The mixed and beef extensive systems of the mountainous, oasis and eastern regions (Fig. 1) (characterised by local breeds traditionally reared) were increasingly marginalised through lack of subsidies, poor access to markets and high transport costs, making it difficult to compete with the emerging dairy sector [29]. The policy was criticised for promoting rural poverty and urban drift, a weak beef cattle sector and poor quality meat. The result was a modern and productive agriculture located in irrigated areas and a more traditional subsistence-oriented agriculture in the less-favourable zones [30].

‘Plan Maroc Vert’ was launched in 2008 to redress this imbalance by supporting small-scale agriculture in deprived areas to help reduce poverty at its roots [31]. The plan, seeking to ‘modernise and homogenise’ agriculture has two pillars: invigoration of high value practice and highly productive farming systems and agro-industry by aggregation of farms (small units grouped into agro-industrial chains) [27] and development of small scale agriculture in marginalised areas [30]. The plan uniquely cuts across different livestock species and production systems.

### Bacteriological evidence

Positive bacteriological isolation is the only incontestable proof of brucellosis and is essential for determination of circulating *Brucella* species and biovars; antibodies are not species specific. Isolation of *Brucella* from live animals uses vaginal discharges from recently aborted animals, milk from lactating females, semen from males or hygroma fluid. Shedding of bacteria is very high in vaginal fluids after abortion; mammary lymph nodes are preferentially colonised leading to frequent milk shedding; males are sentinels of disease by collecting disease from infected females in infected herds, and hygroma fluid is seldom contaminated by other microorganisms (foetuses and placenta, by contrast, are heavily contaminated under local conditions). For necropsied animals, specimens of choice include lymph nodes (mammary, iliac, prefemoral, scapular and cranial), spleen, uterus/epididymides, or mammary glands/accessory sexual glands.

Bacteriological evidence for cattle brucellosis in Morocco is scant (Table 2 and Additional file 1: Table S1). The first isolation of *B. abortus* was from Casablanca in 1965 [32]. The most extensive study [33] examined 500 samples from 357 cattle of imported dairy breeds, yielding 8 *B. abortus* biovar 1 and 28 *B. abortus* biovar 3 strains. Comparison with *B. abortus* strains isolated from cattle in France during the 1980s shows that biotype 1 and 3 were isolated most frequently (778, 28, 793, 17 and 66 strains of *B. abortus* biotype 1, 2, 3, 4 and 9 were isolated respectively) [34]. A subsample (*n* = 12) of Moroccan strains was examined as part of a study characterising 273 *B. abortus* strains of African origin and found the 12 Moroccan strains to be identical to *B. abortus* biotypes isolated from Europe [35]. This was in contrast to strains from sub-Saharan Africa, which showed metabolic differences from both Moroccan (and European) strains [33].

**Table 2:** Summary of brucellosis studies in cattle, small ruminants and humans in Morocco

Study category	No. of studies/reports	Publication type (no. of studies)	Period of studies (no. of studies)	Diagnostic tests (no. of studies)^b^	Sampling (no. of studies)	No. ind (No. of studies)^c^	Range of ind seroprev (%)/incidence	No. herds/flocks (No. of studies)^c^	Range of herd seroprev (%)	References
Cattle						119,288				
Bacteriology	8	T(3), J(5)	1960s(2), 1970s(2), 1980s(3), 2010s(1)	NA	NA	>1007 (6)	NA	NA	NA	[32, 33, 35, 43, 46, 54, 59, 112]
Small-scale serology	11^d^	T(4)^d^, J(4), R(1)	1960s(2), 1970s(4), 1980s(2), 1990s(1), 2010s(2)	SAT/CFT(2), SAT(2), RBT(2), NS(2) RBT/SAT/CFT(1), SAT/RIV(1), RBT/CFT(1)	NPS(8), PS(2), NS(1)	17,548 (11)	0.3–44.3	>256 (6)	2.7–100	[42, 43, 45, 46, 51, 53, 54, 56, 59]^d^
Large-scale serology	4	J(1), R(3)	1970s(1), 1980s(1), 1990s(1), 2010s(1)	SAT/CFT(1), RBT/CFT(1), RBT/CFT/mELISA/MRT(1), RBT(1)	PS?(3), PS(1)	100,733 (4)	2.1–14.1	>4652 (3)	4.6–7.1	[47, 49, 51, 52]
Case reports^a^	23^d^	J(1)^4,^ R(13)	1970s(7), 1980s(3), 2000s(8), 2010s(5)	NS	NPS(23)	NA	2–1505	NA	NA	[44, 113–125]^d^
Small ruminants						55,588				
Bacteriology	3	J(1), R(2)	1990s(3)	NA	NA	>18 (1)	NA	NA	NA	[69–71]
Small-scale serology	10^d^	T(4)^d^, J(5)	1960s(1), 1970s(1), 1980s(3), 2000s(2), 2010s(3)	RBT(6), NS(2), RBT/CFT/RID/DGD/LFA/cELISA(1)	NPS(9), PS(1)	>6810 (9)	0–13.4	>301 (8)	0–50	[45, 54, 79–85]^d^
				RBT/CFT/cELISA(1)						
Large-scale serology	4	J(1), R(3)	1980s(1), 1990s(2), 2000s(1)	RBT/CFT/Coombs(1), RBT/CFT(1), RBT(2)	NPS(1), PS (2), PS? (1)	>48,760 (3)	0–2.5	>831 (2)	0–15.7	[52, 71, 77]
Case reports^a^	13	R(13)	2000s(8), 2010s(5)	NS	NPS (13)	NA	0–25	NA	NA	[113–125]
Humans						4704				
Bacteriology/molecular	5	J(5)	1990s(2), 2000s(1), 2010s(2)	NA	NA	>4 (1)	NA	NA	NA	[90, 97–99, 107]
Serology	3	T(1), J(1), U(1)	1970s(1), 1990s(1), 2010s(1)	SAT(1), RBT(1), SAT/Coombs/RBT/Brucellacapt(1)	NPS (2), PS (1)	4700 (3)	0–3.3	NA	NA	[45, 104]
Case reports^a^	24^d^	T(1)^d^, R(8)^d^	1940s(6), 1950s(3), 1990s(1), 2000s(10), 2010s(4)	NS	NPS (24)	NA	0–42	NA	NA	[45, 126–133]^d^

^a^Number of cases officially reported over a 1 year period

^b^Most studies used either a single serological test or combinations of multiple serological tests in series, with the exception of [47, 54, 77, 80] (see text)

^c^Number of studies which provide information on number of individuals/herds sampled, numerous studies have missing data

^d^Single reference/publication reports multiple studies hence discrepancy with number of studies in category

*T* thesis, *J* journal, *R* report, *U* unpublished, *SAT* serum agglutination test, *CFT* complement fixation test, *RIV* rivanol test, *mELISA* milk ELISA, *MRT* milk ring test, *RID* radial immunodiffusion test, *DGD* double gel diffusion test, *LFA* lateral flow assay, *cELISA* competitive ELISA, *Coombs* coombs test, *NS* not specified, *NA* not applicable, *NPS* non-probability sampling, *PS* probability sampling, *PS?* method poorly described but probability sampling probably applies

Type of samples collected for isolation across studies included aborted foetuses (*n* = 117), placenta (*n* = 161), vaginal discharges (*n* = 519), milk (*n* = 165), hygroma fluid (*n* = 18) and mammary lymph nodes (*n* = 39). Aborted foetuses and placenta are usually heavily contaminated which could explain the low isolation rate in these studies. Few specimens are post-mortem samples suggesting that necropsy is rarely performed as part of brucellosis investigations in Morocco; slaughter of animals is unappealing to farmers in the absence of a compensation scheme.

### Evolution of epidemiological situation and control measures

Most evidence for cattle brucellosis is derived from serological studies (Table 2, Additional file 2: Table S2 and Additional file 3: Table S3), but limitations in the application of serological tests and sampling methodology make such data difficult to interpret. Early studies used the serum agglutination test (SAT), a test lacking sensitivity and specificity [36, 37] and later replaced with the Rose Bengal Test (RBT) and complement fixation test (CFT) used in series The standardisation and origin of RBT is rarely described, which prevents firm conclusions to be reached as inadequate standardisation of RBT results in sensitivity variation between studies [38]. CFT (at a titer 1/4) is a highly specific test in cattle tested 6 months after vaccination but its use as a ‘confirmatory test’ is only warranted in the context of vaccination. Confirmation of RBT positive results is not always required as RBT has excellent sensitivity and specificity (as good as iELISA and other more recently developed smooth lipopolysaccharide or S-LPS tests) in the absence of vaccination [39]. Use of CFT as a confirmatory test should depend on the vaccination status of herds and time elapsed since vaccination, as RBT lacks specificity in vaccinated animals [40]. These technical problems were not considered during serological surveillance pre-dating S19 vaccination in Morocco.

#### 1960s–1987: Emergence of cattle brucellosis in dairy sector

The first serological evidence of cattle brucellosis was seen in the 1960s, 40 years after the first reports of brucellosis in small ruminants (see Brucellosis in small ruminants below) and humans (see Human brucellosis below). Accounts of the situation prior to the 1960s suggest that bovine brucellosis had been a sporadic occurrence until this time, drawing limited State interest [41] (see Emergence in the intensive production system and Extensive production systems below).

Small-scale serological surveys (outbreak investigations) undertaken between 1965 and 1983 indicated brucellosis was an emerging problem in the developing dairy sector (Table 2 and Additional file 2: Table S2). Farms or animals experiencing abortion storms and suspected to have brucellosis were preferentially sampled so the seroprevalence values are likely to have been biased or ‘inflated’ but these studies show a rise in seropositivity over time. Dakkak [42] compared data from annual reports of the brucellosis reference laboratory in Casablanca and observed an increase in seropositivity (SAT and CFT in series) from 8.16 % in 1970 to 15.6 % in 1971. Bekkali [43] returned to the same 35 herds in Rabat (Temara and Ain Aouda) three times over a period of one year and found individual (and herd seropositivity) increased from 15.31 % (45.7 %), to 17.13 % (48.57 %) to 19.67 % (57.1 %). Official case reports from 1973 to 1982 show that up to 1505 cases per year were reported (Table 2 and Additional file 4: Table S4) [44].

Provinces showing high rates of seropositivity were dominated by intensive dairying, especially the industrial urban or agricultural centres of Casablanca, Marrakech, Larache-Tetouan, Meknes, El Jadida, Fes and Rabat [45, 46] (Fig. 1d). These findings were corroborated by the first national survey of 1974–1976 [47, 48] confirming the spread of bovine brucellosis and the need to implement control [48]. A second, more geographically widespread national survey of brucellosis in cattle was commissioned [49] to identify priority regions for control [50]. The 1987–1988 survey showed a similar trend to that described a decade earlier with herd seroprevalence highest in Casablanca (32.86 %), Rabat-Sale (21.15 %), Settat (16.42 %) and Fes (16.35 %)^1^.

#### 1989–1994: National brucellosis control strategy

The results of the 1987–1988 national survey informed a national brucellosis control strategy which adopted a three-tier approach:

- For ‘Group 1’ provinces with a herd seroprevalence of more than 2 % (Casablanca, Rabat-Sale, Taounate, Settat, Fes, Taza, Khemisset, Meknes, Ifrane, Oujda, Khenifra, Benslimane, Tetouan, Boulmane, Tanger, Alhoceima, Sidi Kacem, Nador, Kenitra, El Jadida and El Kella des Sraghna) a program was applied comprising *i*) calfhood S19 vaccination in females 4–7 months old; *ii*) identification of CFT positive males by branding them with letter ‘B’; *iii*) isolation of cows at calving; *iv*) compulsory declaration of abortions and submission of serum and biological samples to brucellosis reference laboratory and *v*) confirmed cases (bacteriologically or serologically) were to be branded with letter ‘B’ [50].
- ‘Group 2’ provinces with an overall herd seroprevalence of less than 2 % (Beni Mellal, Agadir, Taroudant, Marrakesh, Safi, Figuig, Errachidia and Ourzazate) were subjected to a programme of serological surveillance of dairy cattle, bulk milk testing using the ring test (MRT). MRT positive herds were blood sampled to find individual seropositive animals and subjected to the same practices as that for ‘Group 1’. Movement restrictions were imposed on seropositive herds [50].
- ‘Group 3’ provinces, with 0 % herd seroprevalence (Khourigba, Azilal, Chefchouan, Essaouira and the south), were subjected to: *i*) close surveillance of animal movements with neighbouring provinces; *ii*) banned importation of cattle from infected provinces; *iii*) isolation and serological testing of new replacements until confirmation of infection status and *iv*) serological screening of herds every two years [50].

This campaign was badly implemented and failed to reduce the herd seroprevalence in infected regions and to maintain brucellosis free areas. This failure was due to a lack of enforcement of movement restrictions, low levels of S19 vaccination (0.4–8.5 % of the overall heifer population per year) and lack of funds to compensate farmers for culling seropositive animals. Poor vaccination cover was attributed to: an outbreak of African horse sickness and foot and mouth disease which were prioritised by the veterinary services; lack of information on calving period, which resulted in wasted farm visits, compounded by the fact that a lot of herds did not have a calving season and calved all year round necessitating 3 or 4 herd visits to vaccinate all calves; poor organisation of technicians; lack of awareness of brucellosis as a priority problem by livestock keepers and reluctance to collaborate with the veterinary authorities [51].

#### 1990s to present: public-private strategy for control

Despite legislation on brucellosis remaining unchanged (implementation of test-and-slaughter, biosecurity measures, vaccination), this has not been enforced at national level since the failed brucellosis program of the 1990s. The replacement brucellosis program, rolled out in parallel to bovine tuberculosis control, targeted ‘nursery’ farms (‘unite pepiniere’, UP) and farms that are members of professional associations or cooperatives (COPAG- Cooperative Agricole), on a semi-voluntary basis.

The national brucellosis survey of 1996 showed UP farms to be heavily contaminated (individual and herd seroprevalence of 7.1 and 14 % respectively) posing a problem as the legislation stipulates that nursery status is removed upon confirmation of brucellosis infection [51]. The same study found herd seroprevalence in the dairy sector remained high at 6.2 % whereas a small abattoir survey found only 0.25 % of local breed cattle to be seropositive (Additional file 2: Table S2 and Additional file 3: Table S3).

In 2007 COPAG entered into a public-private partnership with the government (ONSSA) and private vets to roll out a brucellosis control strategy for its members. The program put in place test-and-slaughter, culling of reactors, application of strict biosecurity measures and compensation of farmers. There was recognition of the need to educate livestock keepers on the importance of brucellosis control and to ear-tag all cattle on participating farms. Infected herds were serologically tested every 2 months until they had negative results in three consecutive tests. Vaccination was also undertaken on these infected farms: RB51^2^ for serologically negative adult females and S19 for female calves (4–6 months old). Brucellosis free farms were serologically tested every 6 months, and vaccinated with RB51 (adults) and S19 (calves). Between 2007 and 2010, 81,230 cattle were serologically tested, 43,994 and 11,902 cattle were vaccinated with RB51 and S19 respectively and 2901 were culled corresponding to 26 million Dirhams (2.6 million $US) in compensation [52].

Since 2007 the herd seroprevalence for farms signing up to the scheme has reportedly fallen from 40.2 to 0.4 %, although in the absence of information on serological tests and sampling methods it is difficult to draw firm conclusions. The overall national bovine brucellosis seroprevalence remains unchanged: the national survey of 2010–2011 showed an individual and herd seroprevalence of 2.1 and 4.9 % respectively. The herd seroprevalence remained at the same level since 1977 when it stood at 4.6 % or since 1988 when it was at 4.9 % (Additional file 3: Table S3). These data suggest that despite a reduction in brucellosis in UP farms, bovine brucellosis in Morocco has remained unchanged since the 1960s.

Evidence after 2011 is limited to two small-scale serological studies: *(i)* a very high individual seroprevalence (33.48 %, *n* = 221) in 25 herds of Sidi Slimane Province [53]; *(ii)* a cross-sectional survey undertaken in Sidi Kacem (province adjacent to Sidi Slimane) in 2012 showing individual prevalence of 1.5 % (*n* = 1204) [54]. The difference in prevalence between these studies may be due to non-probability methodology used in [53] as compared to a cluster sampling methodology in [54]. The first [53] used the modified protocol of the RBT or mRBT (3:1 ratio of serum to antigen as opposed to 1:1 ratio in standard protocol), shown to have a higher sensitivity for screening of small ruminant sera infected with *B. melitensis* [38, 55], but not validated for use in cattle^3^.

### Emergence in the intensive production system

Even though there are is no quantitative serological evidence on the brucellosis situation in Morocco prior to the 1960s, qualitative accounts based on the observations of early researchers on the origin and spread of the disease are available. Zottner [41] was the first to claim that bovine brucellosis was introduced to the Kingdom through importation of infected cattle from Europe (Netherlands, France, Germany and Poland, countries which at this time were not yet *Brucella* free) [43, 45–47]. Zottner observed that brucellosis outbreaks (abortion storms) across Morocco between 1930 and 1938 could be traced back to contamination by infected imported cattle [41]. Between 1954 and 1970, brucellosis went from being sporadic in a few provinces (estimated seroprevalence of 1 %) to becoming endemic throughout the Kingdom with the exception in the desert zones of the south and southeast. The estimated seroprevalence in 1970 was at 11 %, with peaking at 20 % in certain provinces; abortions were attributed to brucellosis in 37 % of cases [45].

It was over 20 years before the authorities realised the extent to which brucellosis had spread in the country [56] despite regulations being in place to control the infection status of imported cattle, based on serological screening prior to importation [46]. The serological tests available at the time were SAT, later replaced by the then ‘new’ RBT and CFT although there was no evidence that these tests were used for pre-importation screening. None of these tests show 100 % sensitivity, and the diagnostic sensitivity of SAT is lower than that of RBT or CFT [36, 37], particularly in chronically infected herds [57] where many animals have non agglutinating antibodies. Dakkak [42] suggested that brucellosis may have been introduced through the importation of congenitally infected virgin heifers (although the age group most frequently imported was 6 year old cows [45]) as 9 % of congenitally infected heifers do not develop antibodies until the first gestation and are therefore not picked up serologically prior to insemination [58].

The rapid geographical expansion and increase in incidence of bovine brucellosis were attributed to a shift from an extensive livestock production system to a rapidly intensifying mode of dairy cattle production [42, 43, 45, 56]. Disease spread occurred via increases in animal movements across Morocco and the increased frequency of introduction of imported cattle into herds to genetically improve local breeds by cross-breeding (see Cattle production system and policies above) [43]. Brucellosis became established in state owned nursery farms (UP) whose role was to increase the population of European pure and crossbreeds in Morocco to supply other farms. Brucellosis was likely to have been disseminated by redistribution of imported breeds from these nurseries to private farms [56].

Contamination and spread occurred due to the subclinical nature of brucellosis in chronically infected animals, for example by the purchase of infected sexually mature cows from France or Holland, which at 6 years old were obtained for a lower price [45]. The subclinically infected offspring of such cows were then sold to other livestock producers as heifers or cows in calf further spreading brucellosis to recipient farms. Farmers were often unaware of having introduced brucellosis into their herd until transmission to other pregnant females in the herd occurred resulting in an abortion storm.

The growing brucellosis problem in dairy farms remained undetected because no parallel state health programs were instituted, in contrast to schemes to improve nutrition for example, which were thought more important to promote higher productivity. Because of the general lack of veterinary input and monitoring, the state failed to implement control measures in time to halt dissemination [42].

Bekkali [43] found that animals during this period were rarely serologically screened prior to introduction into a herd, and that seropositive cows, rather than being culled (due to lack of state compensation), were often sold on, spreading infection to other farms. Vaccination was illegal in Morocco until 1975, but in the absence of a State led campaign, some livestock keepers covertly vaccinated their cattle with S19. The practice was to vaccinate animals known to be infected, which were sold, further spreading the disease (vaccination does not render an infected animal *Brucella*-free). A further issue with this practice was the serological interference as a result of vaccination, although it was thought that the number of animals vaccinated in this way was minimal [48]. Test-and-slaughter strategy was a sporadic and uncoordinated activity during this period [49].

Early studies saw the process of intensification as the main driver for emergence because of conditions and practices intrinsic to intensive production. Intensive farms acquired heifers and cows in calf of imported breeds produced by the highly infected nursery UP farms. These animals were introduced into the herd without prior serological testing; quarantine of new purchases was not observed; calving boxes were rarely available (separation of animals during calving and in the next weeks that follow minimises exposure) and prolonged suckling increased opportunities for cow to calf transmission of *Brucella* via milk. Poor management practices promoted brucellosis transmission, but increase in herd size was an important factor for the increase in intra-herd prevalence.

### Extensive production systems

Early studies indicate bovine brucellosis in extensively reared autochthonous cattle was largely unknown [46, 56] but the impression was that the disease was rare in these small-scale systems due to animals being reared outdoors [42]. Belkhayat [48], who coordinated the 1974–1976 national survey, considered the role of the traditional system in the epidemiology of brucellosis to be limited, and that outbreaks would quickly ‘die out’ by virtue of the small herd size and the fact that neighbouring herds were far apart and rarely co-mingled. He emphasised the absence or low seroprevalence of brucellosis in Khouribga, Ouarzazate, Khemisset and Safi, where traditional livestock keeping dominated. The protective effect of the mountainous zones, as a physical barrier, may explain failure of the disease to spread to mountainous zones from adjacent infected provinces [49].

The increased susceptibility to brucellosis of imported breeds, compared to local breeds was thought to explain the higher seropositivity in imported stock [42]; a seroprevalence of 44.25 % was found in 504 cattle of imported breed, compared to 4.54 % in 639 cattle of local breed [45]. This view was later refuted on the basis that breed was a confounder for production system, since local breeds dominate the extensive and imported breeds the intensive production system (see Cattle production system and policies) [48]. An outbreak of brucellosis in a herd of 67 native local breed cattle and 140 Friesian cattle maintained on the same farm showed the susceptibility of local breeds [59]. The seropositivity rate in the local cattle 7 months after the first abortion was 80 % and 28 more cows aborted during this period.

The 1987–1988 survey confirmed higher herd seroprevalence in intensive (5.5 %, *n* = 1815) compared to extensive production systems (2.9 %, *n* = 660). Herd size was found to be strongly associated with herd seroprevalence; 2.24, 7.08, 11.23, 11.76 and 15.94 % was reported for herds of 1–10, 11–20, 21–50, 51–100 and >100 cattle respectively. The effect of breed on seroprevalence was also investigated with individual seroprevalence found to be 2.49, 2.53 and 0.27 % for imported, crossbred and local cattle respectively. Overall the survey showed that extensive herds of small size and local breed were less affected by the disease compared to large dairy herds in urban areas.

A recent serological study undertaken in Sidi Kacem province found the irrigated intensive zone dominated by imported breeds had a higher burden of brucellosis (individual seroprevalence 0.3 % [*n* = 602], herd seroprevalence 2.7 % [*n* = 58]) than in the extensive rainfed zone where local and cross-breeds dominate (individual seroprevalence 2.7 % [*n* = 602], herd seroprevalence 10.4 % [*n* = 67]) [54].

Lower transmission is predicted to occur in extensive production systems; many farmers maintain a closed herd and replacement females are rarely introduced into the herd. Cattle in these systems are kept free-range for most of their production cycle and abortions (and birthing) often occur outdoors. *Brucella* does not persist for as long under these conditions because the sun rapidly dries aborted or birthing materials, or stray dogs eat them. Overall lower birth index/longer calving intervals and smaller herd size means that gestation/birthing occurs at lower frequency, reducing opportunities for transmission.

It is argued that pastoralism (especially transhumance) increases the risk of transmission because of the increased opportunity for animals to come into contact with potentially infected herds during their movement and co-mingling [60–64]. Overcrowding of animals during temporary housing or herding in kraals is thought to increase the chance of within-herd transmission; transhumant grazing may allow interaction of wildlife and livestock, facilitating transmission of disease [19].

The evidence would suggest that intra and inter-herd transmission are lower in the extensive than intensive livestock production systems in Morocco. In the absence of control measures, however, smallholder farmers should be considered as a reservoir of disease. This is significant not only from the perspective of potential emergence should the mode of production in these systems intensify, but also because they act as potential reservoirs for re-infection of the intensive livestock sector where brucellosis control is currently focused. The public-private cattle brucellosis control initiative only benefits the intensive and modern sector. The era of the ‘Maroc Vert’ offers great hope for the previously marginalised smallholder systems to catch up with specialised dairy farms. The trade-off from intensification is the potential for emergence of diseases of production [1, 10, 15], and these emerging livestock systems should be closely monitored to prevent brucellosis emergence.

Further bacteriological studies are also required to find whether *Brucella* strains in the local breeds reared extensively are the same as those found in imported breed cattle, or if they are in fact distinct ‘autochthonous’ strains pre-dating the importation of infected cattle from Europe in the 1960s.

## Brucellosis in small ruminants

### Small ruminant production systems and policies

90 % of sheep are reared extensively in rain-fed regions and are located mostly on the high eastern plateau and on western aspects of the middle and higher Atlas (Fig. 2c, d). There are six major sheep breeds found in ‘breed cradle zones’: Timadiht in the middle Atlas; Sardi in the plains north of the Atlas (Chaouia); Beni Guil in the eastern high plateau of the oriental region; Dman of the oases; Beni Hsen in the Gharb and Loukkos regions and Boujaad in the anti-Atlas [65, 66]. Sheep production is declining and nomadic or transhumant systems are being replaced by sedentary systems on irrigated land or near towns. Sheep have a socio-cultural role during Eid El Adha, when around four million are slaughtered [23].

Goats are kept almost entirely under pastoral (rangeland) or sylvopastoral (forest) systems in the mountainous zones of the Atlas in the southwest (Fig. 2e, f). Semi-intensive dairy goats are also kept in the north and oases of the south. Goat breeds have not been genetically characterised, but four main groups are described: dairy breeds of the north, which resemble Spanish breeds (Murcia, Malaguena, Andalouse*);* the middle and higher Atlas hardy breeds; prolific dairy breeds of the oases and a small number of imported breeds (Alpine and Saanen) reared on private and state farms of Chefchaouen and Haouz [67].

The small ruminant sector was neglected prior to the launch of ‘Plan Moutonnier’ in 1980 [66]. This plan partnered government (‘Direction de l’Elevage du Ministere de l’Agriculture et de la Mise en Valeur’) with the ANOC (Association Nationale Ovine et Caprine). The professional association of the ANOC became responsible for delivery of the political programs of the ‘Plan Moutonnier’ to improve the livelihoods of rural-poor small ruminant keepers. 90 % of the activities of the ANOC are focused on a breeding program to maximise meat and milk production, the remaining 10 % focusing on control of parasitic diseases, enterotoxaemia and improvements in animal husbandry. Since the 1990s, the ANOC has carried out organizational, technical and educational activities for farmers [68].

### Bacteriological evidence

Bacteriological evidence of brucellosis in small ruminants is limited to three studies [69–71]. Benhabyles [71] reports isolation of two *B. melitensis* biovar 3 isolates from sheep between 1980 and 1991 but does not describe province of origin of samples (Table 2 and Additional file 5: Table S5). *B. melitensis* biovar 3 was also isolated from sheep in Figuig Province [69] and from small ruminants in Figuig and Jerrada Province (Oriental region, Fig. 1d) in 1996 [70]. These two studies do not describe bacteriological methods used, limiting the conclusions that can be drawn. Further bacteriological studies are clearly required, including in regions where cattle farming dominates. Spill-over of *B. abortus* from cattle to sheep [72–75] and goats [76] has been described in other countries under conditions where these species are co-reared, a common practice in Morocco.

### Evolution of epidemiological situation and Control measures

The serological evidence for small ruminant brucellosis in Morocco is more limited than for cattle (Table 2, Additional file 6: Table S6 and Additional file 7: Table S7).

#### 1921–1990s

The first reported cases of brucellosis in goats originated in Kenitra in 1921, Meknes in 1923–1924, Oujda and Taourit in 1935 and Oulmes in 1936 [46]. Before the emergence of cattle brucellosis in the 1960s, small ruminant brucellosis was the more common form of animal brucellosis in the country [45]. Brucellosis in small ruminants in Morocco remained largely unstudied until the 1990s. Serological surveillance was only undertaken in State-owned goat farms or farms involved in cheese production [45]. Early abortion investigations suggest the presence of brucellosis in Benslimane, Fes, Tetouan, Rabat-Sale for intensively reared goats, and Tetouan, Casablanca and Oujda for sheep [45].

The perception of early researchers was that small ruminant brucellosis in the 1970s was more of a problem in the north Mediterranean zone and inland mountainous areas where small ruminant populations dominated (Fig. 2c–f). Rabat-Sale was considered a hotspot due to the presence of intensively reared Spanish goat breeds. Some authors remarked that Spanish breeds were less resistant to brucellosis than local breeds on account of their over-representation for the disease, but this was more likely due to the intensive mode of production in systems favouring exotic breeds [45].

#### 1980–1996

The first large scale study characterising brucellosis in small ruminants used non-probability sampling methods. The only conclusion that can be drawn is of serological evidence for small ruminant brucellosis in all five regions (north-east, centre north, centre, centre-south and south) during the period 1980–1991 (Additional file 7: Table S7), suggesting brucellosis was endemic in Morocco [71].

In the 1990s, re-emergence of small ruminant brucellosis in the west of Algeria and increasing brucellosis cases in sheep and goats flocks of the Oriental (region adjacent to Algeria) prompted the first probability-sampling survey of small ruminant brucellosis. A survey was undertaken in the Oriental (Fig. 1d) in 1996 by the State (Direction de l’Elevage) covering provinces from the Mediterranean coast to Figuig Province to the south, and showed individual and herd seroprevalence of 2.1 % (*n* = 7771) and 12.1 % (*n* = 628) respectively. The seroprevalence was observed to be increasing from North to South and there was over representation of infected flocks for communes sharing a border with Algeria (Additional file 7: Table S7). Individual seroprevalence was higher in goats than sheep (4.1 and 1.6 % respectively); of 55 mixed (sheep and goat) flocks, 16 (30 %) were considered positive due to presence of at least one positive goat. At this time, there was an increase in importation of goats from Algeria [77] and sheep were also brought from Algeria ‘illegally’, but these were destined for slaughter for the Eid El Adha [70].

In 1997 the state launched a national brucellosis survey to determine the extent of brucellosis spread to other regions and to inform the best strategy and priority regions for control of brucellosis in small ruminants. The results showed that the seropositives detected were from the Oriental region (7 out of 597 animals sampled from Jerrada Province and 46 out of 1565 animals sampled from Figuig Province), suggesting that brucellosis was localised to this zone (Additional file 7: Table S7). Reasons for emergence included: uncontrolled movement of flocks and herds within the region; illegal movements of animals from Algeria as a result of permeability of borders; high frequency of mixed herds (sheep, goats and cattle) and co-grazing of flocks and transhumance (60.7 % of livestock keepers were reported to practise transhumance in the region) [78].

The absence of seropositives in zones other than the Oriental was attributed to the topographical separation of the high eastern plateau from the rest of the country by the middle and higher Atlas and Rif (Fig. 1), making it difficult for animals to be transported beyond these zones^4^. Small-scale surveys undertaken across different regions of Morocco from 1990 onwards, however, suggest that small ruminant brucellosis extends beyond the Oriental. While some studies [54, 79, 80] identified no seropositives in Sidi Slimane, Chefchaouene and Tetouan and Sidi Kacem respectively, others have shown serological evidence of disease in Rabat, Zaers, the middle Atlas and north (Chefchaouene, Tetouan) [81–85] (Additional file 6: Table S6).

These differences could be attributed to differences in sampling methods. Sampling for the national survey consisted of random selection of 1 % of animals turning up at meeting points for goat pox vaccination commissioned by the government; this introduces bias as not all small ruminant keepers turn up for vaccination. The small-scale surveys were abortion investigations in which problem flocks were selected and sampled which would increase the probability of finding seropositives. The results of the 1996 national survey also contradict the 1992 study [71] in which sera screened were stored for years in the freezer at−20 °C and recurrent interruptions of power supply occurred, raising concerns about sera quality.

#### 1996–2003: Vaccination campaign in Oriental

Fikri [78] remarked that the 1996 national survey was too limited in scale to draw any firm conclusions. However, the impression was that urgent State action was needed to prevent brucellosis spread from the Oriental to other zones, and mass vaccination (restricted to the Wilaya of Oujda and Figuig Province) was implemented [86]. FAO recommended that Morocco undertake mass vaccination at national level; resources of the State Veterinary Service were insufficient to scale-up vaccination to this level [87]. Instead all sheep and goats over 3 months old in the Oriental region were vaccinated with Rev 1 (conjunctival route) and vaccination was to be repeated every two years for a minimum of 10 years. On farms co-rearing small ruminants with cattle, cattle over 3 months old were to be vaccinated with S19 via the conjunctival route every 2 years. Vaccination was to be evaluated by serological screening of randomly selected flocks 3 weeks after vaccination (70–100 % of vaccinated animals should be seropositive). Vaccination was to be complemented with serological surveillance at national level to monitor brucellosis spread beyond the Oriental. The impact of the campaign was to be evaluated by collaboration with the public health authorities to monitor the disease in humans [87].

An evaluation of the vaccination campaign was published in 2000 [86]. To achieve adequate coverage vaccination was undertaken every year rather than every 2 years as originally stipulated. Success stories include the rolling out of vaccination in transhumant flocks prior to their transhumance out of the region, preventing dissemination to other zones (such as Taza, Berkane, Fes, Taounate, Fig. 1d). The ‘Direction de l’Elevage’ and ANOC also organised a very successful education campaign to increase receptivity of local farmers to brucellosis vaccination, leading to excellent farmer participation.

Overall, 1,139,225 sheep and 242,180 goat were vaccinated over three years (100 and 88 % of the estimated sheep and goat population respectively) for Oujda Willaya and Figuig Province populations combined [88]. Success in vaccination was reflected in a reduction in official case reports of small ruminant brucellosis between 1997 and 2000.

On average, 404,668 small ruminants were vaccinated per year between 1997 and 2003 [52]. In 1999 a national serological survey (*n* = 13,301) showed 0.6 % individual seroprevalence in Oujda and 0.1 % individual seroprevalence for Morocco as a whole [52] (Additional file 7: Table S7).

Mass vaccination was undertaken in the Oujda, Figuig and Taourirt Provinces up to 2003. The reason for ending vaccination before the recommended 10-year period is unclear. ONSSA claimed that vaccination had improved the epidemiological situation in this region, confirmed by the results of a serological survey undertaken in 2006, which prompted the State to suspend all small ruminant vaccination [89]. The 2006 national survey (*n* = 11,609) yielded 8 seropositives, 5 from the Oriental region and one from each of the Provinces of Khouribga, Al Hoceima and Berkane. ONSSA concluded that these 8 positives could be false positives, which would suggest that small ruminant brucellosis was absent from Morocco [52] (Additional file 7: Table S7). This contradicts official reporting of cases of small ruminant brucellosis since 2002 from Boujdour, Taourirt, Jerrada, Gelmim and Oujda Provinces (Additional file 8: Table S8).

### Potential for re-emergence of small ruminant brucellosis

The small ruminant vaccination campaign of 1996–2003, despite the optimism of the ONSSA did not have a long-term impact on the brucellosis status of the population (small ruminant and human) of the Oriental, and case reports (human [90] and animal) suggest that brucellosis re-emerged and is still present in the region. The campaign curtailed the brucellosis outbreak of 1996, and averted human cases during the period of vaccination. Discontinuation of the vaccination campaign means that more than 10 years on, the whole small ruminant population is once again immunologically naïve and prone to re-infection in the event of emergence of the disease in regions of Algeria sharing a porous border with the region. Unrestricted transportation through open borders has promoted the re-emergence of brucellosis in the Middle East and North Africa region [91].

The reason for prematurely ending the brucellosis vaccination campaign is probably related to changing state priorities but brucellosis control in resource-poor countries requires sustained or long-term mass vaccination [92, 93]. This short-sightedness is not unique to Morocco and has been observed in almost every developing country in the world; governments embark on ambitious, mid- to long-term control programs but these are abandoned as funds are redirected to deal with higher priority emergencies. Two examples are the Greek and Mongolian experience. In Greece, although there was an apparent decrease in small-ruminant abortions and human brucellosis incidence, the infection was still endemic and disease seroprevalence and incidence in animals and humans shot up to return very quickly to initial levels after vaccination was replaced with an uncoordinated test-and-slaughter policy [93]. In Mongolia, mass vaccination undertaken as part of a WHO-endorsed small ruminant brucellosis control campaign was interrupted to deal with a foot and mouth disease outbreak, with subsequent brucellosis recrudescence [94]. FAO/OIE/WHO guidelines for brucellosis prevention and control and the FAO Progressive control program [PCP] could strengthen control in the North Africa region.

## Brucellosis in camels

Pastoralists mainly rear camels in subsistence production systems in the arid Saharan zone (Fig. 1). Despite a substantial camel population in the south of Morocco (Fig. 2g, h), only one study reports detection of *B. abortus*, [45]. Without a description of the diagnostic method (serology versus bacteriology) this information is of limited value; discrimination between *Brucella* species is not possible by serology. Serological and bacteriological studies are required (see Evolution of epidemiological situation below).

## Brucellosis in wildlife

Morocco is home to a range of wild ungulates, which are mostly found in nature reserves situated in all regions of the Kingdom. The most recent population estimates are: 139 dama gazelle (*Gazella dama mhorr*), 550 addax (*Addax nasomaculatus),* 260 Scimitar oryx (*Oryx dammah*), 108 Atlas deer/ Barbary stag (*Cervus elaphus barbarus*), 321 red deer (*Cervus elaphus*), 842 Barbary sheep (*Ammotragus lervia*) [95]. To the best of the authors’ knowledge, no study on brucellosis has ever been undertaken in wildlife in Morocco. Nevertheless, a 0.4 % seroprevalence and isolation of *B. abortus* biovar 1 have been reported in red deer in nearby Spain [96]. Studies on the epidemiology of brucellosis in wild ungulates and their potential role as spillover hosts from or to livestock are lacking.

## Human brucellosis

### Bacteriological and molecular evidence

The only four studies to have isolated and characterised *Brucella* species for human cases report isolation of *B. melitensis* from a man from the south of Morocco [97], a women returning to Taiwan after travel to Morocco [98] and 13 cases diagnosed in France but exposed in Morocco [90, 99] (Additional file 9: Table S9). A Moroccan clinical case report claiming that *B. melitensis* was isolated does not describe the methods for isolation or typing, and cannot be confirmed [97].

Isolation of *B. melitensis* biovar 3 in France from cases exposed in Morocco was reported [90, 99], although only one study adequately describes the typing methods used. *B. melitensis*, whose preferential host is small ruminants, is the main causal agent of human infection in Morocco. *B. melitensis*, especially the predominant biovar 3, constitutes the greatest risk to humans in Mediterranean countries [69, 100, 101]. MLVA analysis revealed that some Moroccan strains cluster with European strains whilst others formed an independent cluster, which suggests that *B. melitensis* may have been imported from Europe but that there could also be local or autochthonous strains [90] .

Transmission from small ruminants to humans is most likely from occupational exposure for livestock keepers, veterinarians and abattoir workers, and by consumption of raw dairy products from small ruminants (especially goats) in the wider population. Consumption of raw goat milk and *jben* (cheese made with raw goat or cow milk) is widespread in Morocco despite the legislation stipulating that goat milk can only be sold by producers with brucellosis free-status [102]. The role of consumption of raw dairy products in transmission is illustrated by additional information gathered on the *Brucella* strains [90]. Seven cases indicated consumption of raw milk or cheese (presumably from goats or sheep although not specified). Human cases of *B. melitensis* as a result of consumption of raw dairy products of cattle origin have been documented [103].

The absence of isolation of *B. abortus* from human cases reflects a lack of studies rather than the relative unimportance of cattle (especially dairy) as a reservoir for human disease. Despite Moroccan legislation imposing pasteurisation of raw milk, informal dairy chains (accounting for 20–30 % of milk consumed) continue to be a hazard for brucellosis transmission [28]. Informal milk chains are active in the dairy basins near large urban centres, as some consumers prefer traditional dairy preparations (*jben*, *raib, smen*, etc.) using raw unpasteurised milk [27]. In a study of 11 rural regions of Morocco, 66 % of households were found to consume raw dairy products [104].

### Evolution of epidemiological situation

There is very little serological evidence on human brucellosis in Morocco (Table 2, Additional file 10: Table S10).

#### 1916–2000s

The first case of human brucellosis in Morocco was reported in El Jadida in 1916, followed by a further two cases in 1922 in Meknes in owners of a dairy goat farm of imported breeds [46]. Other authors claim human brucellosis originated in the Tanger region [56]. 63 alleged cases were reported between 1916 and 1938, prompting the Brucellosis Act for the control of the disease in the animal reservoir [46].

The impression of early researchers was that human cases prior to the mass importation of dairy cattle from Europe in the 1960s were a result of contact with goats or their dairy products, especially those of Spanish breeds reared intensively for milk production. With emergence of brucellosis in cattle, however, *B. abortus* was considered the causal agent of human disease [45]. Prior to mass importation of cattle, human cases were reported from regions with the highest density of goats (Fig. 2e, f), including the Atlas (Azrou, Itzer, Rich, Taza) and the Oriental region (Taourirt, Oujda), whereas later more cases originated from urban centres of Casablanca, Rabat-Sale, Marrakesh, El Jadida, the centres of dairy cattle production (Figs. 1d and 2a, b) [45].

Early researchers were aware of the under-diagnosis and under-reporting of brucellosis [56]. The impression was that Europeans were over represented, as they were more ‘sensitive’ to the disease but it is more likely that Europeans would seek medical attention and be diagnosed. The reluctance of the *Fellah* (Moroccan farmers) to seek medical attention for brucellosis may have resulted in under-detection of the disease [56]. The low rate of disease in local farmers was attributed to farmer practice of selling aborting females (thereby getting rid of the source of contagion), pasteurisation of milk (although recent reports that suggest consumption of raw dairy products is, and always was, widespread in Morocco [27]) and souring of Lben (a yogurt drink) the high acidity of which was wrongly believed to kill *Brucella*^5^.

In 1970 it was found that 2.9 % of 1084 hospital patients in Rabat, Safi, Meknes, Marrakesh, El Jadida, Taza, Tanger and Oujda were serologically positive (SAT titre over 1/80) [45]. Butchers and abattoir workers from Rabat and Kenitra sampled as part of the same study were all seronegative except for one sample that yielded a SAT titre of 1/80 but was CFT negative. SAT is not recommended as a single screening test but the Coombs test performed after SAT detects the non-agglutinating antibodies when they exist so that the SAT-Coombs combination can be used to assess the time of evolution (from high SAT titers and negative Coombs in acute cases to negative SAT and high Coombs titers in long evolution cases). Use of SAT without the Coombs test limits sensitivity. The only conclusion that can be drawn is that human brucellosis was present in this population.

#### 2000s to present

The most ‘comprehensive’ serological study undertaken in Morocco was a three-stage cluster survey conducted on rural populations across 11 regions in 1999 [104]. The study showed an overall seroprevalence of 1.5 % using RBT. Regions most affected were the southern zones of Marrakech -Tensift-El Haouz and Souss-Massa-Draa (Fig. 1d) with 2.8 % and 3.3 % seroprevalence respectively (Additional file 10: Table S10).

A recent hospital study of 593 randomly selected persons presenting to clinics in 2011 in Meknes, Rabat and Kenitra found one person, a 38 year-old woman from Rabat, positive by RBT, SAT/Coombs and Brucellacapt (Ducrotoy et al., unpublished). High Coombs and low SAT titres suggest long evolution brucellosis, but without anamnesis or a clinical history this cannot be confirmed.

The RBT has a high sensitivity (99 %) as its acid pH enables detection of smooth lipopolysaccharide (S-LPS) specific IgM, IgG and IgA and neither prozones nor blocking antibodies are sources of false negative results [105]. The diagnosis of human brucellosis by serology must take into account the fact that some individuals develop antibodies upon exposure to the bacterium but do not become infected, especially in endemic areas [105]. A thorough clinical examination and presence of clinical signs compatible with brucellosis are essential to interpret any brucellosis serological test result, RBT included. In endemic areas, where RBT specificity is likely reduced to 94–96 % [106] weak positive RBT results can be analysed further using the adaptation of the RBT to test serum dilutions. Titres equal to or higher than 1/8 indicate active brucellosis; titres 1/2 and 1/4 must be interpreted according to the presence/absence of symptoms and clinical signs [105].

The rare human cases reported are either diagnosed in referral hospitals of Rabat [97, 107] or were diagnosed abroad (France and Taiwan) [98, 99]. For those diagnosed abroad two scenarios arise: firstly, patients of Moroccan origin presenting to European health services and thereby assumed to have been exposed in Morocco; secondly, patients of non-Moroccan origin with a recent travel history to Morocco presenting to the health services of their home country. Underreporting prevails despite brucellosis being recognised by the authorities as an occupational disease for which compensation can be claimed. Cases are rarely declared because only professionals benefitting from social security can claim compensation [71].

Government reports of brucellosis cases from 1999 onwards indicate between 0 and 27 brucellosis cases reported per year, with a median of 2.5 cases per year (Additional file 11: Table S11). Since 2006, data on the Province of origin has revealed that cases have been reported from the Oriental region (Oujda, Figuig, Jerrada Provinces, Fig. 1d) and the regions of the Sahara (Laayoune, Boujdour, Aoussard, Oued Eddahad Provinces, Fig. 1d). This may reflect a higher index of suspicion for the disease by the health services rather than higher burden of cases in these zones.

Since the outbreak of small ruminant brucellosis in the Oriental region and vaccination campaign, emphasis has been placed on monitoring the brucellosis situation in this region. It makes sense to suspect a higher burden of human disease in a zone where small ruminant brucellosis dominates, as the majority of human cases are caused by *B. melitensis*. The impression of public health experts is that human brucellosis should be most prevalent in the Oriental region [104].

The explanation for the focus of cases from Laayoune (Sahara, Fig. 1d) is unclear. This area has few cattle and small ruminants, but harbours a substantial camel population (Fig. 2g, h), raising questions as to the role of camels as a reservoir of brucellosis. Serological and bacteriological studies on camel and human brucellosis in Laayoune are required.

### Emergence of human brucellosis in Morocco

The recent shift in the distribution of sheep from inland mountainous areas to the coastal plains (Fig. 2c, d) may promote emergence of *B. melitensis*, with consequences for the human population, which consumes large amounts of raw dairy products. Changes in milk consumption habits have also been documented as part of the rural urban drift, and this opens up opportunities for the emergence of brucellosis in humans [27]. Further studies are clearly needed to explore the nature of change in the livestock systems and consumer habits and the impact of this on the dual (animal and human) brucellosis burden. Refai [108] reports that this trend is already a reality in many countries of the Near East region: with recent intensification in importation of animals and establishment of big farms, the incidence of brucellosis has risen sharply in many countries, both in man and animals. Figure 2e, f suggests that the goat population of Morocco is declining, which may also impact on the distribution and prevalence of *B. melitensis* and its transmission to humans.

## Materials and methods

This is a narrative overview of published and grey literature on brucellosis in Morocco as previously tested by Ducrotoy et al. [10].

### Searching

An extensive database search (PubMed, GoogleScholar, Cabdirect) was undertaken using broad search terms in English and French (Brucel* AND Morocco or Brucel* AND Maroc) for 1910 to 30 June 2015. The library database of the Institut Agronomique et Veterinaire Hassan II (IAVHII) [109] was searched in February 2015 using the same search terms to identify veterinary and PhD theses on brucellosis in Morocco. Searches through the references of retrieved articles/theses were also conducted.

Additional data sources were identified through a non-systematic, targeted search of in-country authoritative texts, conference proceedings, personal contacts with experts and unpublished primary research. Data from local seminars, workshops and country reports on brucellosis with limited distribution from 1980 to 2015 were available through Moroccan co-authors. In total, 90 references were identified. Citations were managed in Endnote© reference manager bibliographic software.

### Study selection and inclusion criteria

Of the 90 references, 22 were not obtainable. The first, second and last authors screened the full text of the available 68 articles. Out of 68 studies, 16 were categorised as duplicates (identified by considering author, year of publication, title of paper and comparing abstracts) or as irrelevant (no new epidemiological data was presented, article addresses topic unrelated to the current review such as diagnostic tests or experimental studies) leaving 52 references. Irrelevant articles and justification for exclusion are detailed in Additional file 12: Table S12.

Articles classified as relevant based on screening of full text included two broad categories. Category 1: studies presenting epidemiological data on brucellosis infection in animal and human populations and Category 2: material describing or reviewing strategies employed for brucellosis control in Morocco. Category 1 studies were further subdivided into serological studies, bacteriological studies and official or clinical case reports. General inclusion criteria applicable to both categories included publication date between 1910 and June 2015 and data derived from Moroccan animal or human populations. For serological studies (Category 1), only articles providing some information on the serological test(s) used were included. Secondary sources cited in veterinary theses and local journals for which the original reference was not obtainable were included only when the sampling, population and diagnostic tests were described in sufficient detail.

### Data extraction

**Fig. 3:**
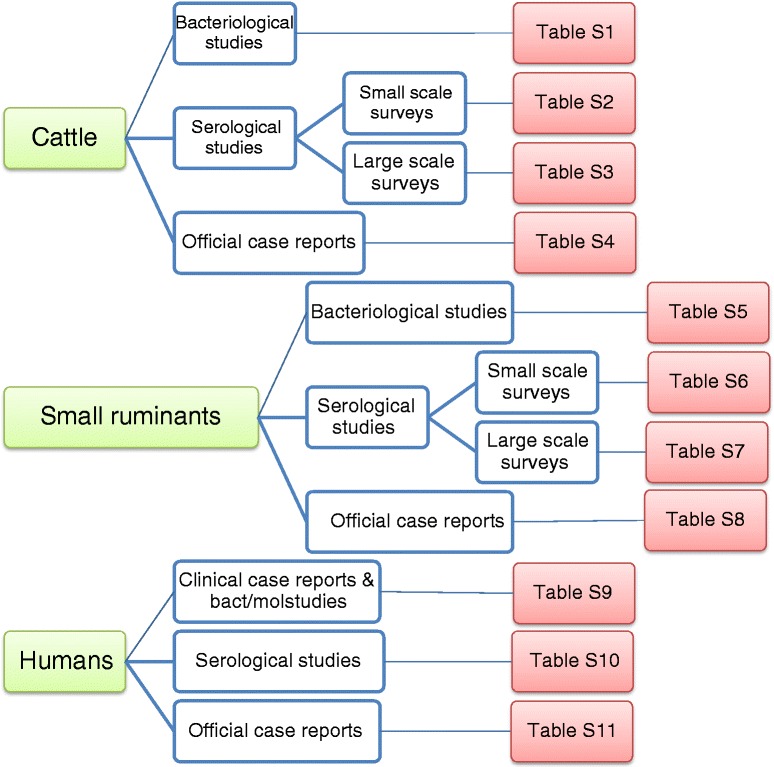
Flow diagram for systematic review of selected studies (bact/mol- bacteriological/molecular)

Category 1 studies were categorised by host (cattle, small ruminants and humans) and grouped into serological studies; bacteriological studies and official case reports (Fig. 3). Serological studies for cattle and small ruminants were subdivided into small-scale surveys at province level and large-scale national or regional government-led surveys (Fig. 3). Extracted data (see Table 3) were firstly synthesised in a Microsoft Excel© database. Data synthesised for Category 1 articles are summarised in Table 2 and Additional file 1: Table S1, Additional file 2: Table S2, Additional file 3: Table S3, Additional file 4: Table S4, Additional file 5: Table S5, Additional file 6: Table S6, Additional file 7: Table S7, Additional file 8: Table S8, Additional file 9: Table S9, Additional file 10: Table S10, Additional file 11: Table S11. Multiple host studies are listed in each of the corresponding summary tables and the common source can be identified by references listed in Additional file 1: Table S1, Additional file 2: Table S2, Additional file 3: Table S3, Additional file 4: Table S4, Additional file 5: Table S5, Additional file 6: Table S6, Additional file 7: Table S7, Additional file 8: Table S8, Additional file 9: Table S9, Additional file 10: Table S10, Additional file 11: Table S11

**Table 3:** Data extracted from studies

Serological surveys	Bacteriological studies	Official case reports
-Population origin -Sampling method (probability versus non-probability sampling) -Sampling approach -Bias or gaps in description of sampling method -Diagnostic test(s) used, cut-off and antigen origin -Location of study -Period of sampling -Sample size -Seroprevalence (individual and herd/flock if available) -Publication type in which data presented (thesis, journal, report)	-Population origin of samples -Region from which samples collected -Period of sampling -Media used for bacteriological culture -Typing methodology -Type and number of biological samples collected -*Brucella* species and biovar isolated -Number of strains isolated -Number of isolates -Publication type in which data presented (thesis, journal, report)	-Year of report Number of cases -Number of outbreaks -Province of origin if data available

Category two studies were categorised by host and read by the main and second author. The synthesised information was complemented and commented by the last author, who led the work on bovine brucellosis epidemiology and control under an FAO project initiated in 1996 and was involved in the coordination of the 1996–2003 small ruminant vaccination campaigns.

### Data analysis

Each paper was reviewed systematically by the main, second and last authors. Additional file 1: Table S1, Additional file 2: Table S2, Additional file 3: Table S3, Additional file 4: Table S4, Additional file 5: Table S5, Additional file 6: Table S6, Additional file 7: Table S7, Additional file 8: Table S8, Additional file 9: Table S9, Additional file 10: Table S10, Additional file 11: Table S11 were created to further summarise data. Quantitative data have not been statistically combined due to methodological heterogeneity (diagnostic tests, sampling approach, study level etc.) of studies.

### Quality assessment

Broad inclusion criteria were applied; even non-probability sampling serological surveys have some value as they provide information on the presence/absence of disease in populations and grey literature can highlight misconceptions surrounding use of diagnostic tests and control approaches. An unpublished study on human brucellosis was included as it was undertaken by the first author who could accurately report methodological detail.

The authors’ interpretation and synthesis of the information gathered is presented taking into account major differences between studies, such as application of probability sampling versus non-probability sampling methods. Serological tests applied varied across studies but the Rose Bengal Test (RBT) was most frequently used (Table 2). Most studies used either a single serological test or combinations of multiple serological tests in series, reporting a single seroprevalence value. There were four exceptions: [47] screened with SAT and CFT in parallel, but reported a single seroprevalence value based on positivity to both tests; [80] and [54] screened sera with a panel of tests but no seropositives were detected therefore a single seroprevalence value (zero) is reported; and [77] using RBT and CFT in parallel, for which we report seroprevalence with regards to RBT positivity for consistency with the national survey conducted by the same author based on the sole use of the RBT [110].

### Maps

Figure 1 displays the elevation, landcover, livestock production/agro-ecological zones and administrative zones of Morocco. The elevation map (Panel A) visualises bathymetric data captured by the Shuttle Radar Topography Mission (SRTM) on a 30 arc second resolution, which roughly corresponds to 1 km. The resulting dataset is available as a worldwide digital elevation model from which data was extracted for Morocco only. The landcover map (Panel B) shows the Land Cover of the World 2000 product, developed within the Global Land Cover 2000 project. The product is based on the VEGA 2000 dataset holding data of 14 months of daily observations by the VEGETATION instrument on board the SPOT 4 satellite. Land cover was classified using the FAO Land Cover Classification System (LCCS). The agro-ecological zones (Panel C) represent eight geographic zones that are characterised by different livestock production practices. In the administrative divisions map (Panel D) both region and province names are given to the administrative units that were valid throughout the livestock survey done in 1996. Small provinces that belong to the same urban centre were aggregated on this map to improve readability.

Maps for ruminant host distribution (Fig. 2) are based on three data sets from the Division of Statistics (DS) of the Moroccan Agricultural Ministry obtained in-country: Demographic data on cattle, sheep, goats and camels from 1996 based on the last national survey; Estimated demographic data on cattle, sheep and goats based on modelled predictions for 2014; and Estimated demographic data on camels based on modelled predictions for 2011.

For provinces in the region of Guelmim-Es-Semara, Laâyoune, Al Haouz, Souss-Massa - Draa and Meknes-Tafilalet these model predictions were aggregated on region level and redistributed among their provinces based on the relative figures in 1996 for which data was available on province level. The 1996 survey data, although dated, is more accurate and complete and is presented alongside the 2011/2014 estimates for comparison (Fig. 2). To create the maps, census data was imported into QGIS 2.6 [111] and joined with a data layer holding the administrative divisions of Morocco on province level as in 1996.

## Conclusion

The evidence reviewed in this paper shows the potential for emergence of brucellosis during a shift from extensive to intensive modes of livestock production and indicates the importance of sustainable control to prevent re-emergence of disease. The review also illustrates challenges faced by Government in obtaining a sound evidence base on which to build control strategies. Huge gaps exist in our knowledge of this important zoonosis in Morocco and livestock seroprevalence values based on probability sampling and incidence in humans are lacking.

The RBT is a cheap and effective test [105], which could be distributed to primary, secondary and tertiary health centres at low cost, enabling health practitioners to screen patients presenting with non-specific symptoms compatible with brucellosis. In this way, additional brucellosis cases to those presenting to referral tertiary hospitals in the capital or health services abroad could be diagnosed and treated.

More bacteriological studies are required to confirm which livestock species and their respective value chains pose a public health risk, as well as the degree of risk associated with specific host species and human habits related to their management and consumption of their products. Such information would be invaluable to target animal reservoir species, practices, operators in the value chain and marketing circuits associated with a high risk of transmission.

Surveillance of human incidence should be a priority for countries such as Morocco where epidemiological data is scarce, as human disease is the best indicator of animal disease. Identifying ‘where’ the human cases are coming from and which animal host species are implicated in transmission can help target control to high-risk livestock systems, which may appeal to policy-makers. In a way, this is what is already being done in Morocco, with control now specifically targeting the intensive dairy farms where brucellosis prevails. Knowledge of trends in human incidence is also important as a proxy measure of the efficiency of control interventions targeting the animal reservoir.

## Additional files

Additional file 1: Table S1. Bacteriological studies for cattle brucellosis. (DOCX 140 kb) Additional file 2: Table S2. Small scale (provincial) serological surveys for cattle brucellosis. (DOCX 139 kb) Additional file 3: Table S3. Large scale government national serological surveys for cattle brucellosis. (DOCX 174 kb) Additional file 4: Table S4. Official case reports of bovine brucellosis per year and province. (DOCX 94 kb) Additional file 5: Table S5. Bacteriological studies for small ruminant brucellosis. (DOCX 52 kb) Additional file 6: Table S6. Small scale (provincial) serological studies for small ruminant brucellosis. (DOCX 138 kb) Additional file 7: Table S7. Large scale (regional and national) serological studies for small ruminant brucellosis. (DOCX 126 kb) Additional file 8: Table S8. Official case reports of small ruminant brucellosis per year and province. (DOCX 61 kb) Additional file 9: Table S9. Clinical case reports and bacteriological and molecular characterisation studies for human brucellosis. (DOCX 86 kb) Additional file 10: Table S10. Serological studies on human brucellosis. (DOCX 105 kb) Additional file 11: Table S11. Number of official case reports of human brucellosis per year and province (if data available). (DOCX 86 kb) Additional file 12: Table S12. Rejected studies. (DOCX 112 kb)

## Abbreviations

ANOC: Association Nationale Ovine et Caprine
CFT: complement fixation test
COPAG: Cooperative Agricole
FAO: Food and Agriculture Organization of the United Nations
GDP: gross domestic product
iELISA: indirect ELISA
mRBT: modified protocol of rose Bengal test
MRT: milk ring test
ONSSA: Office National de Securite Sanitaire des produits Alimentaires
RBT: rose Bengal test
SAT: serum agglutination test
UP: unite pepiniere or nursery farm

## Footnotes

1 The surveys cannot be directly compared due to differences in sampling methodology but data do show a geographic expansion of brucellosis. Most individual prevalence values are fairly consistent across provinces between the two surveys, as is the overall herd prevalence (4.6 % in 1970s compared to 4.9 % in 1980s) (Table S3). The apparent increase in Khemisset (previously a low prevalence area) is likely an artefact due to differences in sample size (800 cattle sampled in 1980s; 450 in 1970s).

2 Controlled experiments have shown RB51 efficiency to be inferior to S19 [134] and RB51 interferes in ELISA and other solid phase assays and so does not necessarily solve the interference of vaccination depending on the serological test used for monitoring. Other drawbacks include the fact that there are no internationally accepted criteria for quality control of RB51; the high cost of RB51 as compared to S19; the fact that no country has eradicated brucellosis using RB51 (except the ‘Azores’, an island) [135]; and the fact that RB51 can cause human infections which are rifampin-resistant.

3 Yahyaoui [54] screened sera in parallel with the standard RBT (sRBT) and modified RBT (mRBT) and found the seropositivity to be much higher with the latter (1.8 and 10.5 % individual seroprevalence for the rainfed and irrigated zones respectively), showing almost a 10 fold increase. Parallel screening of mRBT positive sera with a panel of other serological tests (sRBT, c-ELISA, DGD-NH-double gel diffusion with native hapten-, CFT) suggested that the modified protocol may be ‘oversensitive’ to the detriment of specificity for screening of cattle sera (Ducrotoy, personal data). Further studies with panels of reference positive (from bacteriological positive animals) and reference negative (from animals of Brucella-free areas) are required to investigate the performance of mRBT in cattle further.

4 Transhumance from this zone to the middle Atlas takes place suggesting that the role of transhumance in spreading disease is of less importance [77].

5 It is now recognised that souring of milk does not destroy brucellae as they are preserved in the milk fat. Lactic acid has an inhibitory effect on pathogenic bacteria but this cannot be depended upon to provide a safe milk product.
