# Supplementary material for: Narrative overview of animal and human brucellosis in Morocco: intensification of livestock production as a driver for emergence?
Source: Infect Dis Poverty. 2015 Dec 22;4:57. doi: 10.1186/s40249-015-0086-5 (PMC4687311; doi:10.1186/s40249-015-0086-5)
Supplement: Additional file 1: Table S1. — Bacteriological studies for cattle brucellosis. (DOCX 140 kb) [file 40249_2015_86_MOESM1_ESM.docx]

Table S1 Bacteriological studies for cattle brucellosis

| **Reference** | **Origin of samples** | **Region (n samples)** | **Period of sampling** | **Media** | **Typing** | **Type of samples** | **n** | **Culture positive** | **Isolate** | **Biovar** | **n** | **Comments** |
| --- | --- | --- | --- | --- | --- | --- | --- | --- | --- | --- | --- | --- |
| Chevrier (1963) | NS | Casablanca | 1965 | NS | NS | NS | NS | 12 | *B. abortus* | NS | 12 | Mentioned in Joubert & Fassi Fihri (1966), don’t have original |
| Nguyen & Fassi-Fihri (1968) | Dairy farms of imported cattle | Casablanca, Meknes-Taza | 1966-1967 | Renoux & Gaumont (1966) | CO_2_ dependence, H_2_S production, growth on dyes, urease, monospecific sera | Aborted foetus | 19 | 7 | *B. abortus* | NS | 7 |  |
| Verger & Grayon (1984) | Pie Noire dairy breed | Morocco | 1976-1982 | Alton et al. (1977) | Phage sensitivity, H_2_S production, monospecific sera, growth on dyes, CO_2_ dependence | Placenta, vaginal swab, aborted foetus | NS | 12 | *B. abortus* | biovar 1 | 2 | Strain characteristics identical with those of the *B. abortus* biovars isolated out of Africa (i.e Europe). Same strains as Taoudi et al. (1984) |
|  |  |  |  |  |  |  |  |  |  | biovar 3/6 | 10 |  |
| Taoudi et al. (1984) | Dairy cattle | Rabat-Sale (312), Kenitra-Sidi Kacem (27), Taza-Oujda (18) | 1979-1983 | *Brucella* agar modified (Bio-Merieux) & antiobitics PCB (Bio-Merieux) & 5% horse serum | H_2_S dependence, urease, growth on dyes, phage sensitivity, monospecific sera, oxidative metabolism | Aborted foetus | 54 | 13 | *B. abortus* | biovar 1 | 8 | 500 samples from 357 animals, 47 isolates and 36 strains  Includes strains of Fagouri (1979) and Bekkali (1981) |
|  |  |  |  |  |  | Placenta | 83 | 15 |  |  |  |  |
|  |  |  |  |  |  | Vaginal swab | 264 | 17 |  |  |  |  |
|  |  |  |  |  |  | Milk | 68 | 2 |  |  |  |  |
|  |  |  |  |  |  | Various (hygroma, mammary lymph nodes) | 31 | 0 |  | biovar 3 | 28 |  |
|  |  |  |  |  |  | Aborted fetus, placenta, milk, cervico-vaginal secretions | NS | 2 |  |  |  |  |
| Johson et al. (1984) | Research farm herd of 67 native local cattle and 140 Friesian cattle | Rabat? | 1983 | Alton et al. (1975) | Corbel & Morgan (1975) | Aborted foetus | 35 | 9 | *B. abortus* | biovar 3 | NS | Isolates from stomach content and placental tissues from cows in Friesian herd |
| Fagouri (1979) | Sampled submitted by vets on suspicion of brucellosis. Samples from imported breeds or cross-breeds. | Rabat (194), Sidi Slimane (17), Taza (8), Oujda (6), Ouazzane (2), Fkih ben Salah (4), Kenitra (8) | 1978 | Modified brucella aga enriched with 5-10% serum and antibiotics (PCB) | H_2_S dependence, urease, growth on dyes, phage sensitivity, monospecific sera, oxidative metabolism | Placenta | 56 | 8 | *B. abortus* | biovar 1 | 6 | 277 samples from 239 cattle. 24 isolates and 20 strains from Rabat (7), Sidi Slimane (8), Taza (3) and Oujda (2). 3 additional strains, 2 from Casablanca and 1 from Ksar-El Kebir were donated by the Casa vet lab, yielding a total of 24 strains. 2 biovars have overlapping geographical distribution. |
|  |  |  |  |  |  | Vaginal swab | 135 | 7 |  |  |  |  |
|  |  |  |  |  |  | Milk | 25 | 0 |  |  |  |  |
|  |  |  |  |  |  | Hygroma | 5 | 0 |  |  |  |  |
|  |  |  |  |  |  | Mammary lymph nodes | 18 | 0 |  | biovar 3 | 18 |  |
|  |  |  |  |  |  | Aborted foetus | 5 | 1 |  |  |  |  |
| Bekkali (1981) | 184 samples taken from 90 cattle suspected to have brucellosis | Rabat | 1980-1981 | Modified brucella agar enriched with 5-10% serum and antibiotics (PCB) | H_2_S dependence, urease, growth on dyes, phage sensitivity, monospecific sera, oxidative metabolism | Placenta | 18 | 3 | *B. abortus* | biovar 3 | 10 | 15 isolates and 10 strains |
|  |  |  |  |  |  | Vaginal swab | 116 | 9 |  |  |  |  |
|  |  |  |  |  |  | Milk | 42 | 2 |  |  |  |  |
|  |  |  |  |  |  | Hygroma | 3 | 0 |  |  |  |  |
| Yahyaoui (2012) | RBT positive and aborting cows sampled as part of cross-sectional survey | Sidi Kacem | 2012 | CITA and Farrell’s media | Classical typing (phage sensitivity CO_2_ requirement, H_2_S production, urease, oxidase, monospecific sera, growth on dyes ) and *Brucella* species determined with Bruceladder® PCR | Milk, vaginal swabs | 30 | 3 | *B. abortus* | biovar 1 | 3 | All isolates from same herd of Friesian cattle in intensive irrigated zone. VNTR analysis grouped these with European strains (Ducrotoy, unpublished) |

NS- not specified, PCB- polymixin cyclohexemide bacitracin, CITA- Centro de Investigacion y Technologia Agroalimentaria de Aragon, H_2_S- hydrogen sulphide, CO_2_ –carbon dioxide
