# Supplementary material for: Narrative overview of animal and human brucellosis in Morocco: intensification of livestock production as a driver for emergence?
Source: Infect Dis Poverty. 2015 Dec 22;4:57. doi: 10.1186/s40249-015-0086-5 (PMC4687311; doi:10.1186/s40249-015-0086-5)
Supplement: Additional file 2: Table S2. — Small scale (provincial) serological surveys for cattle brucellosis. (DOCX 139 kb) [file 40249_2015_86_MOESM2_ESM.docx]

Table S2 Small scale (provincial) serological surveys for cattle brucellosis

| **Reference** | **Population** | **Sampling type** | **Sampling method** | **Bias (gaps in method description)** | **Diagnostic test**  **(cut-off/**  **antigen)** | **Period of sampling** | **Province(s)** | **Sample size (no. herds)** | **% Prevalence (herd prev)** | **Comments** |
| --- | --- | --- | --- | --- | --- | --- | --- | --- | --- | --- |
| Joubert & Fassi-Fihri (1966) | Government dairy farms, imported breeds | NPS | Purposive sampling of state herds of imported cattle | Selection criteria of herds not specified | NS | 1965 | Rabat, Casablanca, Tanger, Larache, Oujda, Sidi-Slimane, Souk-el-Arba, Fes, Meknes, Kenitra, Ben-Ahmed, Fedala, El Jadida | 2000 (NS) | 20 (NS) | The purpose of these herds was for milk production and for redistribution of imported breeds to private farms, for cross-breeding and breed improvement |
| Nguyen & Fassi-Fehri (1968) | Dairy cattle, imported breeds predominantly and some local breeds | NPS | Sampling in 50 dairy herds | Selection criteria of herds not specified | SAT (100iu/ml/ NS) & CFT in series* | 1966-1967 | Marrakech | 516 (18) | 15.70 (61.1) | Casablanca sample includes 147 cattle of local breed sampled at abattoir, 4 of which +ve.  *SAT negatives/inconclusives screened with CFT |
|  |  |  |  |  |  |  | Casablanca | 486 (16) | 13.17 (50.0) |  |
|  |  |  |  |  |  |  | Rabat | 412 (12) | 9.95 (33.3) |  |
|  |  |  |  |  |  |  | Larache-Tetouan | 209 (4) | 10.53 (75) |  |
|  |  |  |  |  |  |  | **Overall** | **1623 (50)** | **11.58 (52)** |  |
| Bouatra (1970) | Cattle having aborted | NPS | Samples sent in by vets on suspicion of brucellosis following abortion | Samples tested on suspicion of brucellosis | SAT (100iu/ml/ NS) | 1966-1967 | Casablanca | 30 (NS) | 60 (NS) | This study was undertaken by someone else and is reported in this thesis. The author concludes that approx 40% of abortions are caused by brucellosis. |
|  |  |  |  |  |  |  | Rabat | 55 (NS) | 16.36 (NS) |  |
|  |  |  |  |  |  |  | Marrakech | 12 (NS) | 50 (NS) |  |
|  |  |  |  |  |  |  | Oujda | 7 (NS) | 71.42 (NS) |  |
|  |  |  |  |  |  |  | Meknes-Taza | 30 (NS) | 53.33 (NS) |  |
|  |  |  |  |  |  |  | **Overall** | **134 (NS)** | **40.30 (NS)** |  |
| Bouatra (1970) | Imported and local breeds | NPS | Sera sent to central vet lab by vets | Reason for sera being sent to lab not elucidated (suspected brucellosis case?) | SAT (80iu/ml/ NS) | 1968-1969 | Beni Mellal | 17 (NS) | 0 | This study was undertaken by someone else and is reported in this thesis. |
|  |  |  |  |  |  |  | Berkane | 13 (NS) | 0 |  |
|  |  |  |  |  |  |  | Casa-Port | 1187 (NS) | 0 |  |
|  |  |  |  |  |  |  | Casablanca | 1350 (NS) | 28.67 (NS) |  |
|  |  |  |  |  |  |  | El Jadida | 17 (NS) | 23.53 (NS) |  |
|  |  |  |  |  |  |  | Fes | 105 (NS) | 8.57 (NS) |  |
|  |  |  |  |  |  |  | Kenitra | 261 (NS) | 2.68 (NS) |  |
|  |  |  |  |  |  |  | Marrakech | 131 (NS) | 29.01 (NS) |  |
|  |  |  |  |  |  |  | Meknes | 249 (NS) | 10.04 (NS) |  |
|  |  |  |  |  |  |  | Rabat-Sale | 255 (NS) | 47.45 (NS) |  |
|  |  |  |  |  |  |  | Safi | 5 (NS) | 40.00 (NS) |  |
|  |  |  |  |  |  |  | Settat | 8 (NS) | 25.00 (NS) |  |
|  |  |  |  |  |  |  | Tetouan | 139 (NS) | 6.47 (NS) |  |
|  |  |  |  |  |  |  | **Overall** | **3737 (NS)** | **16.16 (NS)** |  |
| Bouatra (1970) | Imported cattle and local breeds | NS | NS | Method not described | NS | 1970 | Mohamedia, Casablanca, Settat, El Jadida | 504 (19)* | 44.25 (78.9)* | *imported breeds, ** local breeds |
|  |  |  |  |  |  |  |  | 639 (NS)** | 4.54 (NS)** |  |
| Dakkak (1973) | Dairy cattle | NPS | Sera sent to central lab by vets and large dairy enterprises | Sera tested on suspicion of brucellosis | SAT (NS) & CFT (NS) n series | 1970-1971 | Agadir, Beni Mellal, Casablanca, El Jadida, Fes, Kenitra, Khouribga, Marrakech, Meknes, Oujda, Rabat-sale, Settat, Taza | 3282 (NS)* | 8.16 (NS)* | This study was undertaken by someone else and is reported in this thesis, which reviews previous studies.* sera collected 1970, ** sera collected 1971 |
|  |  |  |  |  |  |  |  | 3172(NS)** | 15.6 (NS)** |  |
| Bekkali (1981) | Dairy cattle, 73% imported breeds rest local or cross | NPS | Only animals with history of abortion, placental retention, mastitis, metritis and hygroma sampled | Selection criteria of herds not specified, purposive sampling of females suspected to have brucellosis | RBT (NS), SAT (80 iu/ml/ NS) and CFT (NS) in series | 1980-1981 | Rabat (Temara and Ain Aouda) | 418 (35) | 17.37 (50.46) | The same herds were sampled 3 times over the one year; and the prevalence went from 15.31 (45.7) to 17.13 (48.57) and eventually 19.67 (57.1)% showing emergence of brucellosis in this area. |
| Johnson et al. (1984) | Native local breed cattle kept on same farm as a herd of Friesian cattle | NPS | Outbreak investigation in research herd, sampling of sexually mature animals | One farm investigated for abortion storm | SAT (200iu/ml/ NS) for local breed and RIV (1:50) for Friesian cattle | 1983 | Rabat | 140 (1)* | 14🡺2* (100) | Friesian herd maintained under a calfhood vaccination programme from 1972-1976 when program discontinued. Local breeds never vaccinated. * for Fresians vaccination undertaken and prev reduced. **for local breed no vaccination and prev increased. 🡺change in prev over 4 months of monitoring |
|  |  |  |  |  |  |  |  | 67 (1)** | 3🡺80**(100) |  |
| MAEE (1997) | Abattoir cattle, local breeds only | PS | Random selection of 20% of abattoirs in Morocco (29 in total) and random sampling of 10-15 cows per abattoir | Small sample size, rationale for 20% sampling fraction not elucidated | RBT & CFT in series (Pourquier antigen) | 1996 | NS | 407 (NA) | 0.25 (NA) | Study was a component of the 1996 national survey |
| Yahyaoui (2012) | Local, cross and imported breeds from rainfed and irrigated zones | PS | Cross-sectional survey, cluster sampling | None | RBT (CNBR antigen) | 2012 | Sidi Kacem | 602 (58)* | 0.3 (2.7)* | *Extensive rainfed zone  **Intensive irrigated zones |
|  |  |  |  |  |  |  |  | 602 (67)** | 2.7 (10.4)** |  |
| Lucchese et al. (in press) | Semi-intensive dairy farms | NPS | Purposive sampling; 25 farms selected, herds >11 animals sampling of 10 females over 18 months, herds ≤11 all females over 18 months sampled | Selection criteria of farms not described | mRBT (VLA antigen) | 2014 | Sidi Slimane | 221 (25) | 33.48 (88) | Prevalence seems remarkably high, probably due to using mRBT instead of RBT |

NPS- non-probability sampling, NS-not specified, NA-not applicable, SAT- serum agglutination test, CFT- complement fixation test, RBT- rose Bengal test, RIV- rivanol test, mRBT-modified rose Bengal test, CNBR- Centro Nacional de Referencia para la Brucellosis Granada Spain, VLA- Veterinary Laboratory Agency Weybridge UK.
