# Supplementary material for: Narrative overview of animal and human brucellosis in Morocco: intensification of livestock production as a driver for emergence?
Source: Infect Dis Poverty. 2015 Dec 22;4:57. doi: 10.1186/s40249-015-0086-5 (PMC4687311; doi:10.1186/s40249-015-0086-5)
Supplement: Additional file 3: Table S3. — Large scale government national serological surveys for cattle brucellosis. (DOCX 174 kb) [file 40249_2015_86_MOESM3_ESM.docx]

Table S3 Large scale government national serological surveys for cattle brucellosis

| **Reference** | | **Belkhayat (1976)** | | **MARA (1989a)** | | **MAEE (1997)** | | | | **ONSSA (2013)** | |
| --- | --- | --- | --- | --- | --- | --- | --- | --- | --- | --- | --- |
| **Population** | | Dairy cattle, urban setting | | 1815 INT and 660 EXT herds | | UP or nursery/breeding units (298 farms) | | Dairy farms supplying collection centres (non-UP) (82743 farms) | | UP, non-UP ≤5cattle, non-UP >5cattle | |
| **Sampling type** | | PS? | | PS? | | PS | | PS | | PS? | |
| **Sampling method** | | Random sampling of 15-20% of selected herds | | Sample size per province calculated as 5% of overall pop | | Census, all units sampled | | Simple random sampling applied to 2 sub-groups (farms ≤5 or >5 cattle) | | NS | |
| **Bias/gaps** | | Method for selection of herds not described | | Method for selection of herds not described | | Farms supplying milk to informal sector are not represented | | | | No info on sampling | |
| **Period of samp.** | | 1974-1976 | | 1987-1988 | | 1996 | | | | 2010-2011 | |
| **Diagnostic test (cut-off/antigen)** | | SAT (80iu/ml/NA) and CFT in parallel. Samples positive to both tests considered positive. | | RBT & CFT in series (locally produced antigen) | | RBT & CFT in series (NS) | | herds ≤5: ELISA (ind. milk), RBT & CFT in series; herds >5: MRT (bulk milk) & RBT for MRT +ve farms | | RBT (NS) | |
| **Comments** | | Values are combination of study for samples collected in 1974 (Belkhayat, 1975) then toped-up in 1975-1976 (Belkhayat, 1976) | | Prev for herd size <10 and >100, 2 and 16% | | Vaccination undertaken in 17 and 1% of UP and non-UP farms respectively | | | | Don’t have original data | |
| **Region** | **Province** | **ind n (herd n)** | **Ind prev (herd prev)** | **ind n (herd n)** | **Ind prev (herd prev)** | **ind n (herd n)** | **Ind prev (herd prev)** | **ind n (herd n)** | **Ind prev (herd prev)** | **ind n (herd n)** | **Ind prev (herd prev)** |
| CHAOUIA - OUARDIGHA | Benslimane |  |  | 463 (NS) | 2.6 (8.33) |  |  |  |  | NS (NS) | NS (3.09) |
|  | Khouribga | 390 (NS) | 0 | 739 (NS | 0 |  |  |  |  |  |  |
|  | Settat | 6530 (NS) | 13.03 (NS) | 1211 (NS) | 3.8 (16.42) |  |  |  |  |  |  |
| DOUKKALA - ABDA | El Jadida | 4856 (NS) | 2.41 (NS) | 3404 (NS) | 1.38 (4.81) |  |  |  |  |  |  |
|  | Safi | 981 (NS) | 2.55 (NS) | 1595 (NS) | 0.25 (0.79) |  |  |  |  |  |  |
| FES - BOULEMANE | Boulmane |  |  | 203 (NS) | 7.39 (7.77) |  |  |  |  | NS (NS) | NS (4.44) |
|  | Fes | 3274 (NS) | 13.99 (NS) | 429 (NS) | 6.09 (16.35) | NS (45) | NS (15.5) | NS (45) | NS (15.6) |  |  |
| TAZA - AL HOCEIMA - TAOUNATE | Al Hocema |  |  | 288 (NS) | 1.39 (7.14) |  |  |  |  |  |  |
|  | Taounate |  |  | 156 (NS) | 12.18 (20) |  |  |  |  |  |  |
|  | Taza | 392 (NS) | 3.23 (NS) | 1306 (NS) | 3.11 (10.43) |  |  |  |  |  |  |
| RABAT - SALE - ZEMMOUR - ZAER | Khemisset | 848 (NS) | 1.77 (NS) | 800 (NS) | 5.35 (10.13) |  |  |  |  | NS (NS) | NS (1.35) |
|  | Rabat-Sale | 2089 (NS) | 13.36 (NS) | 751 (NS) | 4.31 (21.15) |  |  |  |  |  |  |
| GHARB - CHRARDA - BENI HSSEN | Kenitra | 6625 (NS) | 2.4 (NS) | 2795 (NS) | 0.76 (5.75) |  |  |  |  |  |  |
|  | Sidi Kacem |  |  | 729 (NS) | 2.63 (6.56) |  |  |  |  |  |  |
| GRAND-CASA. | Casablanca | 2918 (NS) | 23.0 (NS) | 1524 (NS) | 5.77 (32.86) | NS (96) | NS (1.05) | NS (211) | NS (6.1) | NS (NS) | NS (30.77) |
| GUELMIM - ES-SEMARA | Goulmine |  |  |  |  |  |  |  |  | NS (NS) | NS (0) |
|  | Tata |  |  | 287 (NS) | 0 |  |  |  |  |  |  |
| MARRAKECH - TENSIFT - AL HAOUZ | El Kelaa | 1341 (NS) | 2.54 (NS) | 664 (NS) | 0.62 (3.13) |  |  |  |  | NS (NS) | NS (2.02) |
|  | Essaouira |  |  | 878 (NS) | 0 |  |  |  |  |  |  |
|  | Marrakech | 3700 (NS) | 5.03 (NS) | 2209 (NS) | 0.18 (0.8) | NS (39) | NS (38.5) | NS (153) | NS (3.9) |  |  |
| TADLA - AZILAL | Azilal |  |  | 620 (NS) | 0 |  |  |  |  |  |  |
|  | Beni Mellal | 4331 (NS) | 1.87 (NS) | 1912 (NS) | 1.06 (0.49) |  |  |  |  |  |  |
| MEKNES - TAFILALET | Errachidia |  |  | 105 (NS) | 2.91 (9.38) |  |  |  |  | NS (NS) | NS (8.89) |
|  | Khenifra |  |  | 293 (NS) | 5.12 (8.82) |  |  |  |  |  |  |
|  | Meknes-Ifrane | 6085 (NS) | 5.39 (NS) | 785 (NS) | 1.53 (9.84) |  |  |  |  |  |  |
| ORIENTAL | Nador | 235 (NS) | 0 | 1056 (NS) | 2.24 (5.82) |  |  |  |  | NS (NS) | NS (11.01) |
|  | Oujda-Figuig | 1370 (NS) | 6.2 (NS) | 2098 (NS) | 5.33 (9.35) | NS (14) | NS (0) | NS (31) | NS (6.5) |  |  |
| SOUSS - MASSA - DRAA | Agadir | 4378 (NS) | 2.92 (NS) | 866 (NS) | 0.95 (1.85) | NS (35) | NS (8.6) | NS (60) | NS (1.6) | NS (NS) | NS (7.66) |
|  | Ouarzazate | 156 (NS) | 0 | 958 (NS) | 0.55 (0.45) |  |  |  |  |  |  |
|  | Taroudant |  |  | 877 (NS) | 0.13 (1.04) |  |  |  |  |  |  |
|  | Tiznit |  |  | 278 (NS) | 0 |  |  |  |  |  |  |
| TANGER - TETOUAN | Chefchaoun |  |  | 295 (NS) | 0 |  |  |  |  | NS (NS) | NS (3.88) |
|  | Tanger | 735 (NS) | 4.78 (NS) | 867 (NS) | 2.19 (7.55) | NS (69) | NS (2.9) | NS (111) | NS (8.1) |  |  |
|  | Tetouan | 302 (NS) | 1.32 (NS) | 2258 (NS) | 2.78 (8.17) |  |  |  |  |  |  |
| OVERALL | | 51536 (NS) | 8.21 (4.6) | 33699 (2575) | 2.17 (4.9) | 1969 (298) | 14.1 (7.05) | 4538 (611) | NS (6.2) | 8991 (1168) | 2.1 (4.9) |

PS?-method not well characterised but probability sampling probably applies, PS- probability sampling, NS- not specified, RBT- rose Bengal test, CFT- complement fixation test, ELISA- enzyme-linked immunosorbent assay, MRT- milk ring test, UP- unite pepiniere, non-UP- farms non unite pepiniere
