# Supplementary material for: Narrative overview of animal and human brucellosis in Morocco: intensification of livestock production as a driver for emergence?
Source: Infect Dis Poverty. 2015 Dec 22;4:57. doi: 10.1186/s40249-015-0086-5 (PMC4687311; doi:10.1186/s40249-015-0086-5)
Supplement: Additional file 4: Table S4. — Official case reports of bovine brucellosis per year and province. (DOCX 94 kb) [file 40249_2015_86_MOESM4_ESM.docx]

Table S4 Official case reports of bovine brucellosis per year and province [number of cases (number of outbreaks)]

|  | **Sous Massa** | **Jerrada** | **Ben-slimane** | **Sale** | **Berkane** | **El Hajeb** | **Noua-ceur** | **Medi-ouna** | **Sidi Benour** | **Meknes** | **Laay-oune** | **TOTAL** | **Refs** |
| --- | --- | --- | --- | --- | --- | --- | --- | --- | --- | --- | --- | --- | --- |
| **1973** |  |  |  |  |  |  |  |  |  |  |  | 412 | Manes (1984) |
| **1974** |  |  |  |  |  |  |  |  |  |  |  | 1505 | Manes (1984) |
| **1975** |  |  |  |  |  |  |  |  |  |  |  | 1394 | Manes (1984) |
| **1976** |  |  |  |  |  |  |  |  |  |  |  | 98 | Manes (1984) |
| **1977** |  |  |  |  |  |  |  |  |  |  |  | 817 | Manes (1984) |
| **1978** |  |  |  |  |  |  |  |  |  |  |  | 1370 | Manes (1984) |
| **1979** |  |  |  |  |  |  |  |  |  |  |  | 595 | Manes (1984) |
| **1980** |  |  |  |  |  |  |  |  |  |  |  | 59 | Manes (1984) |
| **1981** |  |  |  |  |  |  |  |  |  |  |  | 253 | Manes (1984) |
| **1982** |  |  |  |  |  |  |  |  |  |  |  | 75 | Manes (1984) |
| **...** |  |  |  |  |  |  |  |  |  |  |  |  |  |
| **2002** |  |  |  |  |  |  |  |  |  |  |  | 2 (1) | ONSSA (2002) |
| **2003** |  |  |  |  |  |  |  |  |  |  |  | NS | ONSSA (2003) |
| **2004** |  |  |  |  |  |  |  |  |  |  |  | 72 (2) | ONSSA (2004) |
| **2005** |  |  |  |  |  |  |  |  |  |  |  | 3 (3) | ONSSA (2005) |
| **2006** |  |  |  |  |  |  |  |  |  |  |  | 28 (5) | ONSSA (2006) |
| **2007** | 299 (17) |  |  |  |  |  |  |  |  |  |  | 299 (17) | ONSSA (2007) |
| **2008** |  |  |  |  |  |  |  |  |  |  |  | 45 (1) | ONSSA (2008) |
| **2009** |  | 1 (1) | 36 (4) |  |  |  |  |  |  |  |  | 37 (5) | ONSSA (2009 |
| **2010** |  |  |  | 5 (1) | 8 (1) |  |  |  |  |  |  | 13 (2) | ONSSA (2010) |
| **2011** |  |  |  |  |  | 14 (3) | 2 (1) |  |  |  |  | 16 (4) | ONSSA (2011) |
| **2012** |  |  | 59 (3) |  |  | 10 (1) |  | 15 (1) | 31 (2) | 5 (1) |  | 120 (8) | ONSSA (2012) |
| **2013** |  |  | 6 (1) |  |  |  |  |  | 16 (2) |  | 2 (1) | 24 (4) | ONSSA (2013) |
| **2014** |  |  | 10 (1) |  |  |  | 20 (2) |  |  |  |  | 30 (3) | ONSSA (2014) |
| **Total** | 299 (17) | 1 (1) | 111 (9) | 5 (1) | 8 (1) | 24 (4) | 22 (3) | 15 (1) | 47 (4) | 5 (1) | 2 (1) |  |  |

NS- not specified
