# Supplementary material for: Narrative overview of animal and human brucellosis in Morocco: intensification of livestock production as a driver for emergence?
Source: Infect Dis Poverty. 2015 Dec 22;4:57. doi: 10.1186/s40249-015-0086-5 (PMC4687311; doi:10.1186/s40249-015-0086-5)
Supplement: Additional file 5: Table S5. — Bacteriological studies for small ruminant brucellosis. (DOCX 52 kb) [file 40249_2015_86_MOESM5_ESM.docx]

Table S5 Bacteriological studies for small ruminant brucellosis

| **Reference** | **Origin of samples** | **Region** | **Province (town)** | **Period of sampling** | **Media** | **Biotyping** | **Type of samples** | **n** | **Culture positive** | **Isolate** | **Biovar** | **n** | **Comments** |
| --- | --- | --- | --- | --- | --- | --- | --- | --- | --- | --- | --- | --- | --- |
| Benhabyles et al. (1992) | NS | NS | NS | 1980-1991 | "Brucella modified" or Trypticase soya (Difco) and antibiotic mix (PCB) | As per Alton et al. (1988) | NS | 18 (S) | 2 (S) | *B. melitensis* | Biovar 3 | 2 (S) |  |
| MAMVA (1996) | Bni Guil sheep | Oriental region | Figuig, (Tendrara) | 1996 | NS | NS | NS | NS | NS | *B. melitensis* | Biovar 3 | NS |  |
| El Moudni (1997) | NS | Oriental region | Maatarka (Figuig)  Ain Beni Mather (Jerrada) | 1996 | NS | NS | Products of abortion and vaginal swabs | NS | NS | *B. melitensis* | Biovar 3 | NS | Flock keeper had brucellosis symptoms confirmed as serologically +ve |

NS- not specified, PCB- polymixin, cyclohexidine and bacitracin, S- sheep, G- goats
