# Supplementary material for: Narrative overview of animal and human brucellosis in Morocco: intensification of livestock production as a driver for emergence?
Source: Infect Dis Poverty. 2015 Dec 22;4:57. doi: 10.1186/s40249-015-0086-5 (PMC4687311; doi:10.1186/s40249-015-0086-5)
Supplement: Additional file 6: Table S6. — Small scale (provincial) serological studies for small ruminant brucellosis. (DOCX 138 kb) [file 40249_2015_86_MOESM6_ESM.docx]

Table S6 Small scale (provincial) serological studies for small ruminant brucellosis

| **Ref.** | **Population** | **Sampling method** | **Sampling approach** | **Bias (gaps in method description)** | **Period of sampling** | **Diagnostic test (cut-off/ antigen origin)** | **Province (Region)** | **Sample size (no. flocks)** | | **Positives (no. flocks)** | | **% Prevalence (flock prev)** | | | **Comments** |
| --- | --- | --- | --- | --- | --- | --- | --- | --- | --- | --- | --- | --- | --- | --- | --- |
|  |  |  |  |  |  |  |  | **S** | **G** | **S** | **G** | **S** | | **G** |  |
| Bouatra (1970) | Spanish goat breed (murcienne & grenadine) | NPS | Abortion investigation | Farms selected based on history of abortion | 1964 | NS | Tetouan | NA | 151 (NS) | NA | 20 (NS) | NA | | 13.24 (NS) | Study undertaken by other authors |
|  |  |  |  |  | 1966 |  | Tetouan | 278 (NS) | | 22 (NS) | | 7.91 (NS) | | |  |
|  |  |  |  |  | 1967 |  | Tetouan | 1268 (NS) | | 105 (NS) | | 8.28 (NS) | | |  |
|  |  |  |  |  | 1967 |  | Rabat-Sale | NA | 144 (NS) | NA | 15 (NS) | NA | | 10.42 (NS) |  |
|  |  |  |  |  | 1968 |  | Rabat-Sale | NA | 414 (NS) | NA | 23 (NS) | NA | | 5.56 (NS) |  |
|  |  |  |  |  | 1969 |  | Rabat-Sale | NA | 215 (1) | NA | 4 (1) | NA | | 1.86 (100) |  |
|  |  |  |  |  | 1970 |  | Casblanca-Oujda | 575 (NS) | NA | 15 (NS) | NA | 2.61 (NS) | | NA |  |
|  |  |  |  |  | 1970 |  | Casablanca, Marrakesh, Taza, Sefrou, Settat | NA | 348 (NS) | NA | 10 (NS) | NA | | 2.87 (NS) | Personal study |
| Benkirane et al. (1990) | Mixed breed system | NPS | 10% of flocks sampled including all aborting females, non-aborting females randomly selected | Selection criteria of farms not described, suspect based on abortions | 1989 | RBT (biomerieux*) | Rabat | 304 (23) | NA | 3 (1) | NA | 0.99 (4.35) | | NA | *commercial kit (Brucelloslide test, biomerieux, Lyon) |
| El Idrissi et al. (1995) | Local breeds (Timahdite, Boujaad & Sardi) | NPS | Sampling of 10% minimum of flock, including all aborting females and some randomly selected healthy females | Flocks selected based on report of abortion in last 3 months | 1994 | RBT (biomerieux*) | Zaer and Middle Atlas | 604 (18) | NA | 11 (1) | NA | 1.82 (5.56) | | NA |  |
| Berrada et al. (2004) | Abattoir and farms | NPS | Abattoirs: all adult goats to be slaughtered during day of visit sampled. Farms: s10% of herd sampled including all aborting females | Convenience sampling based on ease of access, cooperation of farmer, herd size and location | 1994 | RBT (NS) | Chefchaouene & Tetouan (North) | NA | 374 (NS)* | NA | 0* | NA | | 0* | * 5 abattoirs  ** 11 herds  Same data as in Hakam et al. (1994) |
|  |  |  |  |  |  |  |  | NA | 81 (11)** | NA | 0** | NA | | 0** |  |
| El Hayany (2005) | Sheep flocks | NPS | Purposive sampling of 'problem flocks', 10 ewes sampled per flock, including all aborting animals | Farms selected based on history of abortion | 2005 | RBT (NS) | Azrou, Ifrane, Boulemane (Middle Atlas) | 235 (22) | | 7 (1) | | 2.98 (4.55) | | | No vaccination in positive flock. Infection blamed on transhumance |
|  |  |  |  |  |  |  |  | 217 (22) | 18 (8) | NS | NS | NS | NS | |  |
| El Idrissi et al. (2005) | Pastoral, agropast. and sylvopast. | NPS | Abortion investigations, 10% of herd/floc sampled including all aborting females and peri-parturient females | Farms selected based on history of abortion | 1997-2000 | RBT (VLA antigen) | Middle Atlas (sylvopastoral), Zaers (agropastoral), Oriental (pastoral), Chefchaouen & Moulay Bouazza (sylvopastoral) | NS (175) | | NS (4) | | NS (2.3) | | | Positives from small ruminants in the Oriental region. |
| Yahyaoui (2012) | Cattle owning farms, rainfed (extensive) and irrigated (intensive) zones | PS | Cross-sectional survey, cluster sampling | Cattle owning households preferentially selected | 2012 | RBT/mRBT (CNRB antigen), CFT, RID, DGD-NH, LFA, c-ELISA | Sidi Kacem | 1031 (NS) | 51 (NS) | 0 | 0 | 0 | | 0 | Goat screened with RBT and mRBT only. Results presented here refer to lab re-screening undertaken by Ducrotoy (unpublished) rather than data presented in thesis |
| Ammary (2014) | 21 sheep flocks | NPS | Purposive selection of flocks with abortion problem, sampling of females having aborted | Farms selected based on history of abortion | 2013-2014 | RBT/mRBT (CNRB antigen), CFT, c-ELISA | Sidi Slimane | 194* (21) | NA | 0 | NA | 0 | | NA | * only 20 of 194 samples tested with RBT and mRBT |
| Benkirane et al. (2015) | Females from farms reporting abortions | NPS | Convenience sampling of 20 sera from each flock/herd including from all primiparous females and all aborting females | Farms selected based on history of abortion | 2013 | mRBT (VLA antigen) | Chefchaouene & Tetouan (North) | 202 (13) | 106 (10) | 27 (5) | 14 (5) | 13.37 (38.4) | | 13.21 (50) |  |

NPS- non-probability sampling, NS- not specified, NA- not applicable, RBT- rose Bengal test, mRBT- modified rose Bengal test, CFT- complement fixation test, c-ELISA- competitive ELISA, RID- radial immunodiffusion test, DGD-NH- double gel diffusion with native hapten, LFA- lateral flow assay, VLA- Veterinary Laboratory Agency Weybridge UK, CNRB- Centro Nacional de Referencia para la Brucellosis Granada Spain. S- sheep, G- goats
