# Supplementary material for: Narrative overview of animal and human brucellosis in Morocco: intensification of livestock production as a driver for emergence?
Source: Infect Dis Poverty. 2015 Dec 22;4:57. doi: 10.1186/s40249-015-0086-5 (PMC4687311; doi:10.1186/s40249-015-0086-5)
Supplement: Additional file 7: Table S7. — Large scale (regional and national) serological studies for small ruminant brucellosis. (DOCX 126 kb) [file 40249_2015_86_MOESM7_ESM.docx]

Table S7 Large scale (regional and national) serological studies for small ruminant brucellosis

| **Reference** | **Population** | **Sampling method** | **Sampling approach** | **Bias (gaps in method description)** | **Period of sampling** | **Diagnostic test (cut-off) (antigen origin)** | **Province/region** | **Sample size (no. flocks)** | | | **Positives (no. flocks)** | | | **% Prevalence (flock prev)** | | **Comments** |
| --- | --- | --- | --- | --- | --- | --- | --- | --- | --- | --- | --- | --- | --- | --- | --- | --- |
|  |  |  |  |  |  |  |  | **S** | **G** | | **S** | | **G** | **S** | **G** |  |
| Benhabyles et al. (1992) | Bank of small ruminant sera collected during three periods 1) 1980-1983, n=66; 2) 1984-1987, n=1780; 3)1988-1991, n=1365. Total sera 3211 | NPS | Purposive selection of 'problem' flocks as part of on-going study on small ruminant health, sampling of 10% of flock, including all females that had aborted either during last lambing season or since | Sampling prompted by health issues | 1980-1991 | RBT, CFT and coombs in series (NS) | Taza, Fez, Oujda, Figuig | NS (83) | NS (13) | | NS (15) | | NS (3) | NS (18.07) | NS (23.08) | Study prompted by suspicion that small ruminant brucellosis emerging in regions sharing a border with Algeria |
|  |  |  |  |  |  |  | Kenitra, Khemisset, Rabat-Sale, Skhirat-Temara | NS (43) | NS (17) | | NS (2) | | NS (2) | NS (4.76) | NS (11.76) |  |
|  |  |  |  |  |  |  | Settat, Ben Slimane, El Jadida, Casablanca | NS (9) | NS (5) | | NS (4) | | NS (0) | NS (44.44) | NS (0) |  |
|  |  |  |  |  |  |  | Beni-Mellal, Khenifra | NS (8) | NS (3) | | NS (1) | | NS (0) | NS (12.50) | NS (0) |  |
|  |  |  |  |  |  |  | Agardir, Ouarzazate | NS (4) | NS (18) | | NS (1) | | NS (1) | NS (25.00) | NS (5.56) |  |
|  |  |  |  |  |  |  | **Overall** | **NS (147)** | **NS (56)** | | **NS (23)** | | **NS (6)** | **NS (15.65)** | **NS (10.71)** |  |
| El Idrissi et al. (1997) | Sheep and goats in oriental region, 6442 sheep and 1329; 429 mixed flocks, 187 sheep flocks and 12 goat flocks | PS | Stratified sampling according to geo-ecological zones, focusing on sheep flocks | Sampling frame may not be exhaustive as not all farms registered | 1996 | RBT & CFT in parallel (Institut Pourquier) | Berkane-Taourirt, Oujda-Angad, Jerrada, Figuig (Oriental) | 7771 (628) | | | 161 (76) | | | 2.07 (12.1) | | Survey prompted by increasing reports of brucellosis cases in the Oriental.  RBT +ves reported for consistency with national survey |
|  |  |  |  |  |  |  |  | 6442 (NS) | | 1329 (NS) | 106 (67) | 55 (9) | | 1.6 (NS) | 4.2 (NS) |  |
| El Idrissi et al. (1997) | National flock | PS | Sampling during goat pox vaccination. Random selection of 10% of meeting points for vaccination in country, sampling of 1% of small ruminants > 1yo that turn up for vaccination | Population brought to vaccination points may be bias sub-sample of overall population | 1997 | RBT (Institut Pourquier) | Centre | 2487 (NS) | | 570 (NS) | 0 | | | 0 | | *Berkane, Taourirt, Nador, Taza & Oujda Provinces all -ve, Jerrada 7 out of 597 +ve (1.2%). Figuig Province 46 out of 1565 (2.9%). Positive provinces share border with Algeria. |
|  |  |  |  |  |  |  | Centre-North | 1688 (NS) | | 224 (NS) | 0 | | | 0 | |  |
|  |  |  |  |  |  |  | Centre-South | 1408 (NS) | | 101 (NS) | 0 | | | 0 | |  |
|  |  |  |  |  |  |  | North-West | 2523 (NS) | | 325 (NS) | 0 | | | 0 | |  |
|  |  |  |  |  |  |  | Oriental | 1673 (NS) | | 441 (NS) | 52 (NS)* | | | 2.46 (NS)* | |  |
|  |  |  |  |  |  |  | South | 1115(NS) | | 682 (NS) | 0 | | | 0 | |  |
|  |  |  |  |  |  |  | Tensift | 2405 (NS) | | 437 (NS) | 0 | | | 0 | |  |
| ONSSA (2013) | NS | PS? | NS | NS | 1999 | NS | NS | 13301 (NS) | | | 19 (NS) | | | 0.14 (NS) | | Supposed post-vaccination assessment. Positives: 7 from Oujda and 1 from Casablanca, 5 sheep and 3 goat samples. Authors conclude these are false positives and speculate Morocco is free of *B. melitensis.* |
|  | NS | PS? | NS | NS | 2006 | RBT (NS) | NS | 11609 (NS) | | | 8 (NS) | | | 0.07 (NS) | |  |

NPS- non-probability sampling, PS- probability sampling, NS- not specified, RBT- rose Bengal test, CFT- complement fixation test, S- sheep, G- goats
