# Supplementary material for: Narrative overview of animal and human brucellosis in Morocco: intensification of livestock production as a driver for emergence?
Source: Infect Dis Poverty. 2015 Dec 22;4:57. doi: 10.1186/s40249-015-0086-5 (PMC4687311; doi:10.1186/s40249-015-0086-5)
Supplement: Additional file 8: Table S8. — Official case reports of small ruminant brucellosis per year and province. (DOCX 61 kb) [file 40249_2015_86_MOESM8_ESM.docx]

Table S8 Official case reports of small ruminant brucellosis per year and province [number of cases (number of outbreaks)]

|  | **Boujdour** | **Taourirt** | **Jerrada** | **Guelmim** | **Oujda Angad** |  |  | **TOTAL** | **Refs** |
| --- | --- | --- | --- | --- | --- | --- | --- | --- | --- |
| **2002** |  |  |  |  |  |  |  | 0 | ONSSA (2002) |
| **2003** |  |  |  |  |  |  |  | NS | ONSSA (2003) |
| **2004** |  |  |  |  |  |  |  | 11 (1) | ONSSA (2004) |
| **2005** |  |  |  |  |  |  |  | 21 (2) | ONSSA (2005) |
| **2006** |  |  |  |  |  |  |  | NS | ONSSA (2006) |
| **2007** |  |  |  |  |  |  |  | NS | ONSSA (2007) |
| **2008** |  |  |  |  |  |  |  | NS | ONSSA (2008) |
| **2009** | 25 (2) | |  |  |  |  |  | 25 (2) | ONSSA (2009) |
| **2010** |  |  | 6 (1) |  |  |  |  | 6 (1) | ONSSA (2010) |
| **2011** |  |  | 16 (1) |  |  |  |  | 16 (1) | ONSSA (2011) |
| **2012** |  |  |  | 1 (1) |  |  |  | 1 (1) | ONSSA (2012) |
| **2013** |  |  |  |  | 25 (1) |  |  | 25 (1) | ONSSA (2013) |
| **2014** |  |  |  |  |  |  |  | NS | ONSSA (2014) |
| **Total** | 25 (2) | | 22 (2) | 1 (1) | 25 (1) |  |  |  |  |

NS- not specified
