# Supplementary material for: Narrative overview of animal and human brucellosis in Morocco: intensification of livestock production as a driver for emergence?
Source: Infect Dis Poverty. 2015 Dec 22;4:57. doi: 10.1186/s40249-015-0086-5 (PMC4687311; doi:10.1186/s40249-015-0086-5)
Supplement: Additional file 9: Table S9. — Clinical case reports and bacteriological and molecular characterisation studies for human brucellosis. (DOCX 86 kb) [file 40249_2015_86_MOESM9_ESM.docx]

Table S9 Clinical case reports and bacteriological and molecular characterisation studies for human brucellosis

| **Reference** | **Origin of samples** | **Region** | **Period of sampling** | **Media** | **Typing** | **Type of samples** | **n** | **Culture positive** | **Isolate** | **Biotype/biovar** | **n** | **Comments** |
| --- | --- | --- | --- | --- | --- | --- | --- | --- | --- | --- | --- | --- |
| Blain et al. (1997) | Moroccan man presenting to Paris hospital with thoracic aneurysm | Morocco | 1996 | NS | NS | Swab of aortal wall, thrombus, blood | 4 | 4 | *Brucella melitensis* | biovar 3 | 3 | Patient also had spondylitis  SAT titre 1/1280 |
| El Hassani et al. (1998) | Cases of primary psoas abscess presenting to three hospitals in Rabat | Morocco | 1987-1997 | NS | NS | NS | NS | NS | NS | NS | NS | SAT was strongly positive in 2 out of 16 psoas abscess cases |
| Ennibi et al. (2009) | Man diagnosed with hepatic brucelloma at Hopital militaire et d'instruction Mohammed-V, Rabat | South of Morocco | 2008 | NS | NS | Blood | NS | NS | *Brucella melitensis* | NS | NS | RBT positive |
| Chuang et al. (2011) | Woman returning to Taiwan after travel to Algeria and Morocco with thoracic aortic embolism and lumbar discospondylitis | Morocco and Algeria | 2010 | aerobic culture bottles (BacT/ALERT, bioMerieux Inc.) | Vitek GN ID system, confirmation by analysis of partial 16S rRNA gene sequencing and comparison with published GenBank sequences | Blood | NS | NS | *Brucella melitensis* | NS | NS | Patient had close contact with camels and ate cheese and milk whilst in desert |
| Lounes et al. (2014) | Cases diagnosed in France, exposure in Morocco | Morocco | 1996-2014 | NS | CO_2_ requirement, H_2_S production, urea hydrolysis, agglutination with monospecific sera, due sensitivity and phage typing | NS | NS | NS | *Brucella melitensis* | biovar 3 | 14 | All cases (apart from one lab-acquired infection) are Moroccan expats living in France or French persons who had visited Morocco and consumed raw dairy products. No info on geographical origin of strains. Authors state that strains form two distinct clusters, suggesting existence of lineage resulting from socio-historical connections between North Africa and Europe. |

NS- not specified, SAT- serum agglutination test, RBT- rose Bengal test, CO_2_- carbon dioxide, H_2_S- hydrogen sulphide
