# Supplementary material for: Narrative overview of animal and human brucellosis in Morocco: intensification of livestock production as a driver for emergence?
Source: Infect Dis Poverty. 2015 Dec 22;4:57. doi: 10.1186/s40249-015-0086-5 (PMC4687311; doi:10.1186/s40249-015-0086-5)
Supplement: Additional file 10: Table S10. — Serological studies on human brucellosis. (DOCX 105 kb) [file 40249_2015_86_MOESM10_ESM.docx]

Table S10 Serological studies on human brucellosis

| **Reference** | **Population** | **Sampling type** | **Sampling method** | **Bias (gaps in method description)** | **Diagnostic test (cut-off)** | **Period of sampling** | **Region/City** | **Sample size** | **% Prevalence** | **95% CI**  (calculated using cluster sampling) | **Comments** |
| --- | --- | --- | --- | --- | --- | --- | --- | --- | --- | --- | --- |
| Bouatra (1970) | Hospital patients (men, women and children) | NPS | Patients with conditions other than brucellosis selected | Patient selection not described | SAT (> 1/80) | 1970 | Rabat, Safi, Meknes, Marrakech, El Jadida, Taza, Tanger, Oujda | 1084 | 2.86 | NA |  |
|  | Butchers and abattoir workers |  | NS | Selection of individuals not described |  |  | Kenitra, Rabat-Sale | 153 | 0 | NA | 1 serum had 1/80 SAT titre but was found CFT negative |
| Belahcen et al. (1999) | Rural population | PS | Three-stage cluster sample; random selection of province, followed by commune, followed by locality. 25 individuals randomly selected from 120 localities. Persons over 3 years eliligible for sampling. | Sampled size based on pre-determined feasibility of collecting 3000 samples | RBT | 1999 | Taza-Al Hoceima-Taounate | 293 | 0 | 0.0-3.0 | Prevalence was found to be highest in the Sahara (Sous-Massa Draa) and Marakesh region, which goes against the hypothesis that brucellosis prevalence should be higher in the Oriental region (prev 1.4 by comparison), although 95% CI similar so cannot draw firm conclusions |
|  |  |  |  |  |  |  | Chaouia-Ourdigha | 205 | 0.5 | 0.0-5.1 |  |
|  |  |  |  |  |  |  | Doukkala-Abda | 274 | 1.1 | 0.1-5.0 |  |
|  |  |  |  |  |  |  | El Gharb-Chararda-Bni Hssen | 323 | 1.2 | 0.2-4.7 |  |
|  |  |  |  |  |  |  | Fes-Boulmane | 75 | 0 | 0.0-11.0 |  |
|  |  |  |  |  |  |  | Marrakesh-Tensift-El Haouz | 425 | 2.8 | 1.2-6.2 |  |
|  |  |  |  |  |  |  | Meknes-Tafilalet | 225 | 0.4 | 0.0-4.7 |  |
|  |  |  |  |  |  |  | Region Orientale | 221 | 1.4 | 0.2-6.2 |  |
|  |  |  |  |  |  |  | Souss-Massa Daraa | 429 | 3.3 | 1.5-6.8 |  |
|  |  |  |  |  |  |  | Tadla-Azilal | 200 | 0 | 0.0-4.4 |  |
|  |  |  |  |  |  |  | Tanger-Tetouan | 200 | 2.5 | 0.6-8.2 |  |
|  |  |  |  |  |  |  | **Overall** | **2870** | **1.5** | **1.0-2.3** |  |
| Ducrotoy et al. (unpublished) | Hospital patients | NPS | Random sampling of patients turning up to hospital | Hospitals selected based on hypothesised presence of WNV in hospital catchment area for WNV study ^[[1]](#footnote-1)^ | SAT/Coombs  RBT  Brucellacapt | 2011 | Meknes | 150 | 0 | NA | * single positive sample: SAT <20; Coombs anti-IgG 160; Coombs anti-IgA 640; Brucellacapt 320. High Coombs and low SAT titre suggests long evolution case but in absence of anamnesis or knowledge of symptoms difficult to interpret. |
|  |  |  |  |  |  |  | Rabat | 268 | 0.37* | NA |  |
|  |  |  |  |  |  |  | Kenitra | 175 | 0 | NA |  |

NPS- non-probability sampling, PS- probability sampling, NS- not specified, SAT- serum agglutination test, RBT- rose Bengal test, WNV- West Nile Virus

1. El Rhaffouli, H., El Harrak, M., Lotfi, C., El Boukhrissi, F., Bajjou, T., Laraqui, A., Hilali, F., Kenfaoui, M., Lahlou-Amine, I., 2012. Serologic evidence of West Nile virus infection among humans, Morocco. Emerg Infect Dis 18, 880-881. [↑](#footnote-ref-1)
