# Supplementary material for: Narrative overview of animal and human brucellosis in Morocco: intensification of livestock production as a driver for emergence?
Source: Infect Dis Poverty. 2015 Dec 22;4:57. doi: 10.1186/s40249-015-0086-5 (PMC4687311; doi:10.1186/s40249-015-0086-5)
Supplement: Additional file 11: Table S11. — Number of official case reports of human brucellosis per year and province (if data available). (DOCX 86 kb) [file 40249_2015_86_MOESM11_ESM.docx]

Table S11 Number of official case reports of human brucellosis per year and province (if data available)

|  | **Oujda** | **Figuig** | **Jerrada** | **Laa-youne** | **Boujdour** | **Aou-ssard** | **Oued Eddahab** | **TOTAL** | **Refs** |
| --- | --- | --- | --- | --- | --- | --- | --- | --- | --- |
| **1940** |  |  |  |  |  |  |  | 2 | Bouatra (1970) |
| **1941** |  |  |  |  |  |  |  | 10 | Bouatra (1970) |
| **1942** |  |  |  |  |  |  |  | 10 | Bouatra (1970) |
| **1943** |  |  |  |  |  |  |  | 5 | Bouatra (1970) |
| **1944** |  |  |  |  |  |  |  | 2 | Bouatra (1970) |
| **1947** |  |  |  |  |  |  |  | 3 | Bouatra (1970) |
| **1951** |  |  |  |  |  |  |  | 42 | Bouatra (1970) |
| **1954** |  |  |  |  |  |  |  | 34 | Bouatra (1970) |
| **1957** |  |  |  |  |  |  |  | 23 | Bouatra (1970) |
| **…** |  |  |  |  |  |  |  |  |  |
| **1999** |  |  |  |  |  |  |  | 0 | DELM (1999) |
| **2000** |  |  |  |  |  |  |  | 0 | DELM (2000) |
| **2001** |  |  |  |  |  |  |  | 0 | DELM (2001) |
| **2002** |  |  |  |  |  |  |  | 2 | DELM (2002) |
| **2003** |  |  |  |  |  |  |  | 1 | DELM (2003) |
| **2004** |  |  |  |  |  |  |  | NS | DELM (2004) |
| **2005** |  |  |  |  |  |  |  | 0 | (DELM 2005) |
| **2006** |  |  |  | 5 |  |  |  | 5 | DELM (2013) |
| **2007** |  |  |  | 24 | 1 | 1 | 1 | 27 | DELM (2013) |
| **2008** |  |  |  | 3 | 1 |  |  | 4 | DELM (2013) |
| **2009** | 18 |  |  | 3 |  |  |  | 21 | DELM (2013) |
| **2010** | 5 | 9 |  | 1 |  |  |  | 15 | DELM (2013) |
| **2011** | 1 | 1 | 14 |  |  |  |  | 16 | DELM (2013) |
| **2012** | 1 |  |  |  |  |  |  | 1 | DELM (2013) |
| **2013** | 3 |  |  |  |  |  |  | 3 | DELM (2013) |
| **Total (1999-2013)** | 28 | 10 | 14 | 36 | 2 | 1 | 1 | 95 |  |

NS- not specified
