# Supplementary material for: Narrative overview of animal and human brucellosis in Morocco: intensification of livestock production as a driver for emergence?
Source: Infect Dis Poverty. 2015 Dec 22;4:57. doi: 10.1186/s40249-015-0086-5 (PMC4687311; doi:10.1186/s40249-015-0086-5)
Supplement: Additional file 12: Table S12. — Rejected studies. (DOCX 112 kb) [file 40249_2015_86_MOESM12_ESM.docx]

Table S12 Rejected studies

| **Reference** | **Species** | **Publication type** | **Study description/title** | **Reason for rejection** |
| --- | --- | --- | --- | --- |
| Azizi (2010) | All | Thesis | Qualitative knowledge, attitudes and practices (KAP) study on brucellosis in Sidi Kacem Province as part of ICONZ project | No new epidemiological data (serological or bacteriological) |
| Benkirane (2001) | NA | Journal | Review of general principles and different strategies recommended for the epidemiological surveillance and control of brucellosis in cattle and small ruminants, with particular reference to the region of North Africa and the Near East | No new epidemiological data (serological or bacteriological) |
| Benkirane (2006) | All | Journal | Ovine and caprine brucellosis World distribution and control eradication strategies in West Asia North Africa region | No new epidemiological data (serological or bacteriological) |
| Bouzagou (1983) | Cattle | Thesis | Vaccination experiment using reduced dose of S19 in dairy herd of 150 cattle and herd of 80 local cows | Vaccine study, no epidemiological data and same data as El Hraiki (1984) |
| Doumbia ((2012) | Camels | Thesis | Rev1 vaccination study in camels | No epidemiological data generated or presented |
| El Hraiki (1984) | Cattle | Thesis | Vaccination experiment using reduced dose of S19 in dairy herd of 200 cattle and herd of 80 local cows | Vaccine study, no epidemiological data |
| El Idrissi et al. (2001) | Small ruminants | Journal | Comparison of efficacy of RB51 and Rev 1 against experimental infection in pregnant ewes | Vaccine study, no epidemiological data |
| El Marrakchi & Hamama (1996) | Goats | Thesis | Assessment of hygienic quality of goat’s cheese | No brucellosis data presented |
| Embarki (2010) | All | Thesis | Review of brucellosis and BTB and small-scale qualitative KAP study amongst Sidi Kacem veterinarians as part of ICONZ project | No new epidemiological data (serological or bacteriological) |
| Fallahi (2014) | All | Thesis | Elaboration and evaluation of community education intervention on brucellosis and BTB in Sidi Kacem as part of ICONZ project | No new epidemiological data (serological or bacteriological) |
| Gwida et al. (2010) | Cattle, small ruminants, camel | Journal | Review of brucellosis studies from North Africa, Middle East and India | No data for Morocco |
| Hotez et al. (2012) | NA | Journal | Review of prevalence, distribution and opportunities for control of NTDs in Middle East and North Africa | No data for Morocco |
| Khalid (1996) | All | Thesis | Literature review of animal and human brucellosis in Morocco | No new epidemiological data and studies cited could not be traced nor corroborated by Moroccan co-authors of the paper. |
| Mennane et al. (2007) | Cattle | Journal | Assessment of hygienic quality of raw cow's milk | No brucellosis bacteriology undertaken |
| Refai (2002) | All | Journal | Review of literature on brucellosis for Near East region | No new epidemiological data (serological or bacteriological) |
| Renoux and Renoux (1975) | Humans | Journal | Serological survey | Unclear if samples are from Morocco or France |

NA- not applicable
